# Synergism and Subadditivity of Verbascoside-Lignans and -Iridoids Binary Mixtures Isolated from *Castilleja tenuiflora* Benth. on NF-κB/AP-1 Inhibition Activity

**DOI:** 10.3390/molecules26030547

**Published:** 2021-01-21

**Authors:** Luis David Arango-De la Pava, Alejandro Zamilpa, José Luis Trejo-Espino, Blanca Eda Domínguez-Mendoza, Enrique Jiménez-Ferrer, Leonor Pérez-Martínez, Gabriela Trejo-Tapia

**Affiliations:** 1 Centro de Desarrollo de Productos Bióticos, Instituto Politécnico Nacional, Yautepec, Morelos 62731, Mexico; larangod1300@alumno.ipn.mx (L.D.A.-D.l.P.); jtrejo@ipn.mx (J.L.T.-E.); 2 Centro de Investigación Biomédica del Sur, Instituto Mexicano del Seguro Social, Xochitepec, Morelos 62790, Mexico; enriqueferrer_mx@yahoo.com; 3 Centro de Investigaciones Químicas, Universidad Autónoma del Estado de Morelos, Cuernavaca, Morelos 62209, Mexico; bed@uaem.mx; 4 Instituto de Biotecnología, Universidad Nacional Autónoma de México, Cuernavaca, Morelos 62210, Mexico; leonor@ibt.unam.mx

**Keywords:** AP-1, *Castilleja tenuiflora*, NF-κB, Orobanchaceae, pharmacodynamic interactions, RAW-blue™ cells

## Abstract

Pharmacodynamic interactions between plant isolated compounds are important to understand the mode of action of an herbal extract to formulate or create better standardized extracts, phytomedicines, or phytopharmaceuticals. In this work, we propose binary mixtures using a leader compound to found pharmacodynamic interactions in inhibition of the NF-κB/AP-1 pathway using RAW-Blue™ cells. Eight compounds were isolated from *Castilleja tenuiflora*, four were new furofuran-type lignans for the species magnolin, eudesmin, sesamin, and kobusin. Magnolin (60.97%) was the most effective lignan inhibiting the NF-κB/AP-1 pathway, followed by eudesmin (56.82%), tenuifloroside (52.91%), sesamin (52.63%), and kobusin (45.45%). Verbascoside, a major compound contained in wild *C. tenuiflora* showed an inhibitory effect on NF-κB/AP-1. This polyphenol was chosen as a leader compound for binary mixtures. Verbacoside-aucubin and verbascoside-kobusin produced synergism, while verbascoside-tenuifloroside had subadditivity in all concentrations. Verbascoside-kobusin is a promising mixture to use on NF-κB/AP-1 related diseases and anti-inflammatory *C. tenuiflora*-based phytomedicines.

## 1. Introduction

*Castilleja tenuiflora* Benth. is a hemiparasite plant from the Orobanchaceae family and is widely distributed throughout Mexican territory, especially in pine-oak forests. Traditionally, *C. tenuiflora* has been used in Mexico to treat the symptoms of cancer, sterility, gastrointestinal disorders, cirrhosis, inflammation, and respiratory diseases in the form of tea or infusions of different parts of the plant [1,2]. The pharmacological properties of *C. tenuiflora* have been related to the presence of plant secondary metabolites (PSM), such as glycosylated iridoids: aucubin, geniposidic acid, bartsioside, 8-epiloganin, mussaenoside, carioptoside, and geniposide [3]; flavonoids: apigenin and luteolin 5-methyl ether [4]; phenylethanoids: verbascoside and isoverbascoside [5]; and lignans: tenuifloroside [4]. Methanolic extracts from wild *C. tenuiflora* collected from different populations (Juchitepec and Parque Nacional Lagunas de Zempoala, Mexico), displayed different pharmacological activities such as cytotoxic [6], antioxidant [6,7], anti-inflammatory [3,8], antiulcerogenic [8], antidepressant [4,9], and sedative [4]. However, these effects were not consistent with the collection site. Methanolic extracts obtained from wild *C. tenuiflora* plants of Parque Nacional Lagunas de Zempoala, containing verbascoside and aucubin [3], displayed cytotoxic activity [6], whereas extracts containing these compounds obtained from wild plants of Juchitepec did not show cytotoxic activity against the same cell lines [8], even if verbascoside and aucubin are cytotoxic by themselves [10,11]. Thus, it was hypothesized that pharmacological interactions could affect the pharmacological activity of *C. tenuiflora*. Pharmacological interactions occur when the pharmacological effect of one drug or PSM is altered by that of another drug or PSM [12]. Pharmacological interactions can be pharmacokinetic, which is when they are related with the changes in the absorption, distribution, metabolization, and elimination of active principles or pharmacodynamic when they work together to affect different targets or the same targets, leading to additive, synergistic, or subadditive actions [13]. Pharmacodynamic additivity is the sum of the pharmacological effects of each individual pharmacological agent in the combination. Synergy occurs when the overall effect of the drug combination is greater than additive and sub-additivity occurs when the drug combination’s effect is less than additive [12]. Pharmacodynamic interactions can be desirable, as seen in synergism between actein and digitoxin, where actein improves the growth inhibitory effect of digitoxin on human breast cancer cells through the inhibition of Na^+^–K^+^-ATPase activity [14]; the verbascoside and 5-Fluorouracil synergistic cytotoxic interaction significantly reduces PI3K and the p-AKT/total AKT ratio and causes G1 cell cycle arrest in colorectal cells [15]. Pharmacodynamic interactions can also be undesired, like the subadditive interaction between loreclezole and clonazepam in the fixed ratio 3:1 with pentylenetetrazole, which induced seizures in mice [16].

The inflammatory process is involved in most chronic degenerative diseases, such as cancer [17], Alzheimer’s [18], atherosclerosis [19], pneumonia [20], rheumatoid arthritis [21], and diabetes [22], among others. It is regulated by several mediators that are produced after the activation of different transcription factors, such as nuclear factor kappa B (NF-κB) and the activator protein 1 (AP-1), which are related to the production of proinflammatory cytokines [23]. Therefore, inhibition of NF-κB and AP-1 has been the molecular target in the search and design of anti-inflammatory drugs. RAW-Blue cells are derived from murine macrophages with chromosomal integration of a secreted embryonic alkaline phosphatase (SEAP) reporter gene which is inducible by NF-κB and AP-1 through TLR-4 signaling. Thus, LPS-stimulated murine RAW-Blue cells have been used to identify anti-inflammatory compounds and to investigate the anti-inflammatory signaling pathway of plant extracts and isolated compounds [24,25].

Binary mixtures using a leader compound are proposed to study pharmacodynamic interactions. These consist of a leader compound with known pharmacologic activity and at least one kind of major PSM (terpenes and phenolic compounds), which are present in methanolic extracts of *C. tenuiflora.* Verbascoside was proposed as a leader compound as it is a common secondary metabolite in wild *C. tenuiflora* plants with anti-inflammatory and NF-κB/AP-1 inhibitory activity [26]. The objective of this study was to determine the presence of pharmacodynamic interactions in mixtures of verbascoside-iridoids and verbascoside-lignans from *C. tenuiflora* on anti-inflammatory activity mediated by NF-κΒ/ΑP-1 using RAW-Blue™ cells and binary mixtures as experimental approach. Study of pharmacodynamic interactions contributes to the understanding of the biological activities of plant extracts and lays the groundwork for developing novel targeted therapies based on chemical compounds derived from medicinal plants. This is the first time that non-glycosylated furofuran lignans are described for *C. tenuiflora,* as well as the pharmacodynamic interactions between verbascoside with aucubin and lignans (tenuifloroside and kobusin). Further, we present experimental evidence of the possible anti-inflammatory mechanism of action of *C. tenuiflora* extract through NF-κΒ/ΑP-1 inhibition besides COX-2 [27].

## 2. Results

### 2.1. Isolation and Identification of Compounds

**Figure 1:**
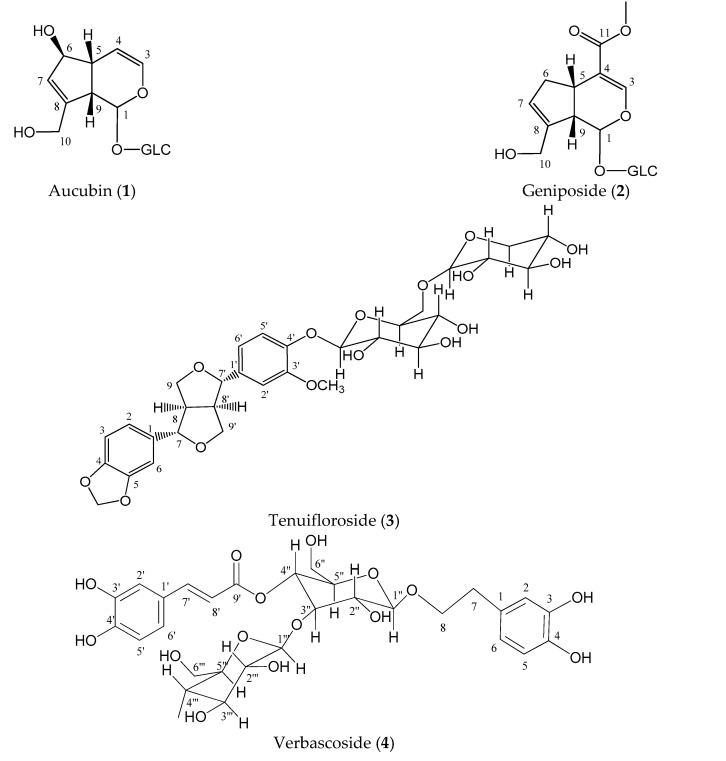

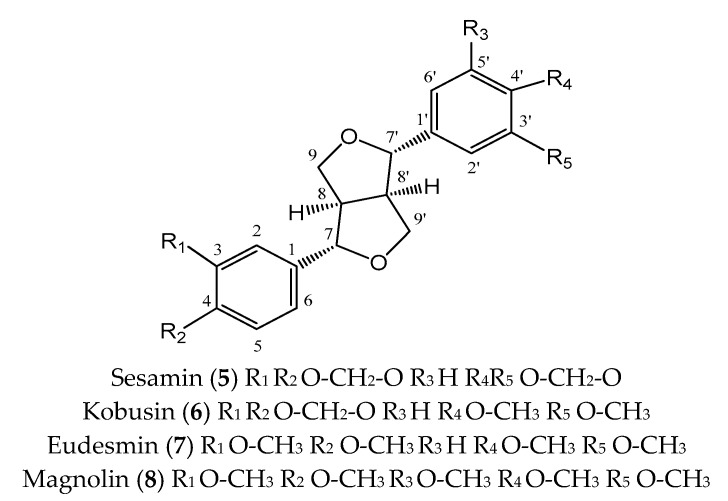
Major compounds isolated from *Castilleja tenuiflora* methanolic extract.

The chromatographic process of methanolic extract from aerial parts of *C. tenuiflora* allowed the isolation of eight different compounds (Figure 1). According to UV spectra, retention time and mass spectra, peak 2 corresponded with geniposide, compound 3 with tenuifloroside and 4 with verbascoside. Compounds 1, 5, 6, 7, and 8 were identified using ^1^H- and ^13^C-NMR.

Compound 1: (Aucubin) ^13^C-NMR (400 MHz, CDCl_3_) δc (ppm): 148.1 (8), 141.6 (3), 130.5 (7), 105.9 (4), 100.1 (1′), 97.9 (1), 97.9 (11), 82.9 (6), 78.3 (5′), 78.0 (3′), 75.3 (2′), 71.7 (4′), 63.1 (6′), 61.8 (10), 48.1 (9), 46.6 (5). ^1^H-NMR (400 MHz, CDCl_3_) δc (ppm): Aglycone 6.3 (dd, *J* = 6.2, 6.2 Hz, 1H), 5.7 (s, br, 1H), 5.1 (dd, *J* = 5.8, 6.2 Hz, 1H), 4.9 (d, *J* = 6.9 Hz 1H), 4.44 (dd, *J* = 3.6, 1.8 Hz, 1H), 4.3 Ha (d, *J* = 15.4 Hz, 1H), 4.1 Hb (d, *J* = 15.4 Hz, 1H), 2.9 (t, *J* = 7.3, 1H), 2.6 (m, 1H). Glucose: 4.6 (d, *J* = 8.1 Hz, 1H), 3.8 Ha (dd, *J* = 12.1, 11.7 Hz, 1H), 3.6 Hb (dd, *J* = 11.3, 11.3 Hz, 1H), 3.4 (t, *J* = 8.8 Hz, 1H), 3.3 (m, 1H), 3.2 (m, 1H), 3.2 (dd, 11.3, 9.1 Hz, 1H) (Figures S19 and S20). According to UV spectra, the absence of a signal greater than 160 ppm corresponding to C = O in ^13^C-NMR was expected. This is corroborated by the ^13^C-NMR spectrum. In *C. tenuiflora*, two iridoids have been reported with these characteristics, aucubin and bartsioside, the difference between them is the presence of an OH at the C6 in aucubin. The signal at 82.9 ppm belonged to CH-OH, confirming the presence of aucubin. Experimental data was compared with that reported by Ersoz et al. [28]. ESI [M + H]^+^: 347.48 *m*/*z* (Figure S9).

Compound 5: (Sesamin) ^13^C-NMR (400 MHz, CDCl_3_) δc (ppm): 147.9 (4,4′), 146.7 (3,3′), 134.9 (1,1′), 119.2 (6,6′), 108.1 (5,5′), 106.4 (2,2′), 101.1 (O-CH_2_-O), 85.7 (7,7′), 71.6 (9,9′), 54.2 (8,8′). ^1^H-NMR (400 MHz, CDCl_3_) δc (ppm): 6.8 (s, br, 1H), 6.8 (s, br, 1H), 6.8 (s, br, 1H), 5.9 (s, 2H,), 4.7 (d, *J* = 4.1 Hz, 1H), 4.2 Hb (dd, *J* = 7.0, 9.3 Hz, 1H), 3.8 Ha (dd, *J* = 3.5, 9.3 Hz, 1H), 3.1 (1H, m) (Figures S21 and S22). It had a typical UV spectrum of furofuran lignans. The furofuran-type lignans have a base skeleton of 18 carbons but in ^13^C-NMR only 10 signals were observed. This indicates that the molecule is symmetric of 20 carbons. Signals corresponding to CH_3_-C and C=O were not observed, but the signal corresponding to R-O-CH_2_-O-R’ (101.9 ppm, 5.9 (s)) was present. Sesamin is a symmetric furofuran lignan with methylenedioxy and experimental data match that reported by Calle [29]. ESI [M + Na]^+^: 378.16 *m*/*z* (Figure S11).

Compound 6: (Kobusin) ^13^C-NMR (400 MHz, CDCl_3_) δc (ppm): 149.9 (3′), 148.4 (4′), 147.7 (3), 147.56 (4), 134.9 (1), 134.9 (1′), 119.1 (6), 118.1 (6′), 110.9 (5′), 109.1 (2′), 107.9 (5), 106.3 (2), 100.9 (O-CH_2_-O), 85.7 (7), 85.6 (7′), 71.6 (9,9′), 55.8 (8′), 54.2 (4a’) 54.1 (3a’), 53.9 (8). ^1^H-NMR (400 MHz, CDCl_3_) δc (ppm): 6.9 (d, *J* = 1.8, 1H), 6.85 (d, *J* = 1.7, 1H), 6.8 (s, 1H), 6.8 (s, 1H), 5.9 (s, 2H), 4.7 (d, *J* = 4.9 Hz, 1H), 4.7 (d, *J* = 4.7, 1H), 3.9 (s, 3H), 3.8 (s, 3H), 3.1 (m, 1H), 3.1 (m) (Figures S23 and S24). Compound 6 presented 21 signals in the ^13^C-NMR spectrum similar to that of sesamin, thus, the molecule was not symmetric. A single signal could be observed at 100.86 ppm, evidencing that one of the methylene-di-oxy groups was open and the signals of 54.1 ppm and 54.2 ppm (3.8 s) were methoxy groups. Experimental data correlated to kobusin [30]. ESI [M + Na]^+^: 394.57 *m*/*z* (Figure S15).

Compound 7: (Eudesmin) ^13^C-NMR (400 MHz, CDCl_3_) δc (ppm): 149.1 (3,3′), 148.6 (4,4′), 133.5 (1,1′), 118.2 (6,6′), 111.0 (5,5′), 109.21 (2,2′), 85.7 (7,7’), 71.7 (8-8′), 55.9 (9-9′), 55.9 (3a-3a’), 54.1 (4a-4a’). ^1^H-NMR (400 MHz, CDCl_3_) δc (ppm): 6.9 (s, br, 2H), 6.8 (s, br, 2H), 6.8 (s, br, 2H), 4.7 (d, *J* = 3.1 Hz, 2H), 4.2 (m, 2H), 3.9 (s, 6H), 3.8 (m, 2H), 3.8 (s, 6H), 3.1 (s, br) (Figures S25 and S26). Eleven signals were observed in the ^13^C-NMR spectrum. As sesamin, compound 7 was also symmetric. The R-CH_2_-O-CH_2_-R’ signal was absent but O-CH_3_ signals were observed at 55.9 ppm (3.9 (s) ppm) and 54.1 ppm (3.8 (s) ppm). Chemical shifts coincided for eudesmin [31]. ESI [M + Na]^+^: 410.87 *m*/*z* (Figure S10).

Compound 8: (Magnolin) ^13^C-NMR (400 MHz, CDCl_3_) δc (ppm): 153.4 (3′), 153.4 (5′), 149.2 (4), 148.6 (3), 137.4 (4′), 136.8 (1), 133.4 (1′), 118.2 (6), 111.0 (5), 109.2 (2′), 102.8 (2), 102.8 (6′), 86.0 (7), 85.7 (7′), 71.9. (9), 71.7 (9′), 60.8 (4a’), 56.2 (5a’), 55.9 (3a’), 55.9 (3a), 55.9 (4a), 54.4 (8′), 54.1 (8). ^1^H-NMR (400 MHz, CDCl_3_) δc (ppm): 6.9 (d, *J* = 1.83 Hz), 6.8 (d, *J* = 1.83 Hz), 6.8 (s), 6.8 (s), 6.5 (s), 4.7 (d, *J* = 4.5 Hz), 4.7 (d, *J* = 4.5 Hz), 3.9 (3H, s), 3.9 (3H, s), 3.8 (3H, s), 3.8 (3H, s), 3.8 (3H, s), 3.1 (m), 3.1 (m) (Figures S27 and S28). Compound 8 was an asymmetric lignan. A 102.8 ppm signal was found for R-CH_2_-O-CH_2_-R’ but the absence of a signal in 6.0 ppm of ^1^H-NMR showed that the methylene-di-oxy group was not present. There was also the presence of signals corresponding to methoxy groups (50–55 ppm). Compared to eudesmin, there were differences between 102.8 and 153.4 ppm, corresponding to the aromatic regions, indicating an extra substituent. An extra methoxy in C5′ explains the changes in the aromatic region and the signal in 153.4 ppm. Experimental data match with magnolin, according to that reported by Miyazawa et al. [32]. ESI [M + Na]^+^: 440.13 *m*/*z* (Figure S14).

### 2.2. EC_50_ and E_max_ of the Isolated Compounds

It was observed that none of the previously isolated compounds in the concentration range of 0.01–100 and 0.1–200 µM for verbascoside were toxic against RAW-Blue ™ cells (Figure S29). Therefore, the ability of the compounds to inhibit NF-κB/AP-1 activity was evaluated in those concentration ranges. All isolated compounds inhibited NF-κB/AP-1 and their EC_50_ (effective concentration 50) and E_max_ (maximal effect) were calculated (Table 1).

### 2.3. Pharmacodynamic Screening Using Binary Mixtures

Verbascoside-iridoids and verbascoside-lignans binary mixtures were evaluated looking for a combination with possible presence of pharmacodynamic interactions. So, first it was necessary to corroborate EC_50_ determination. Solutions of each compound at its EC_50_ were prepared and evaluated in RAW-Blue™ cells, where slight variations were observed, confirming that the determination was accurate (Table S1). Corroborated EC_50_ were considered to calculate the expected NF-κB/AP-1 inhibition (%) of each binary mixture. Expected NF-κB/AP-1 inhibition was the inhibition activity calculated considering additivity between the corroborated inhibition at EC_50_ of verbascoside and the corroborated inhibition at EC_50_ of the different iridoids and lignans (Table S1). Binary mixtures were carried out combining each compound with verbascoside at its EC_50_. Verbascoside-iridoids mixtures had similar NF-κB/AP-1 inhibition and inhibition ratio with respect to expected data (Table 2). Verbascoside-aucubin and verbascoside-geniposide mixtures inhibited NF-κB/AP-1 by 46.16% and 43.04%, respectively, with an inhibition ratio of 0.84 and 0.89, respectively.

Verbascoside-lignans mixtures showed a wide range of NF-κB/AP-1 inhibition and inhibition ratios. Verbascoside-kobusin showed the highest NF-κB/AP-1 inhibition (63.06%) and inhibition ratio (1.13) of all binary mixtures, followed by verbascoside-eudesmin (51.01%, 0.97), verbascoside-sesamin (47.40%, 1.05), and verbascoside-magnolin (39.33%, 0.74), whereas verbascoside-tenuifloroside showed the lowest values (37.99%, 0.74).

Verbascoside-kobusin and verbascoside-tenuifloroside binary mixtures were selected to look for pharmacodynamic interactions among verbascoside-lignan mixtures because the inhibition ratio value suggested synergism and subadditivity; verbascoside-aucubin was chosen to aim for verbascoside-iridoids pharmacodynamic interactions, despite verbascoside-geniposide presenting a slightly better inhibition ratio, aucubin was easier to isolate than geniposide.

### 2.4. Pharmacodynamic Interactions

Inhibition ratios of the selected binary mixtures were obtained at different EC_50_ proportions. The verbascoside-aucubin mixture presented synergism at 0.75EC_50_/0.25EC_50_ and 0.50EC_50_/0.50EC_50_. In contrast, at 0.25EC_50_/0.75EC_50_, this mixture presented subadditivity (Table 3). NF-κB/AP-1 inhibition enhanced when verbascoside concentration increased in relation to aucubin.

Conversely, verbascoside-kobusin showed synergism at all concentrations. The mixture at equal proportion of each compound (0.50EC_50_/0.50EC_50_) presented the highest NF-κB/AP-1 inhibition activity (%) and inhibition ratio of all mixtures: 43.23% and 1.56, respectively. In contrast, verbascoside-tenuifloroside mixtures displayed subadditivity in all concentrations, whereas the 0.50EC_50_/0.50EC_50_ verbascoside-tenuifloroside mixture had the highest subadditive effect (14.16%, 0.46). Binary mixtures with verbascoside as leader compound allowed to find pharmacodynamic interactions.

## 3. Discussion

Herbal extracts are very complex matrices of different PSM. Most of the time, pharmacological activity is attributed to the major PSM of the herbal extract or standardized fraction. However, the possibility that the PSM present in the herbal extract may work together at different parts and levels of a signaling pathway, in this case, the NF-κB/AP-1 pathway, must be considered. In this work, we tried to understand how PSM, present in the methanolic extract, may work together to inhibit NF-κB/AP-1. For this purpose, it was necessary to determine the presence of pharmacodynamic interactions between the main constituents of *C. tenuiflora* found in the methanolic extract.

*Castilleja tenuiflora* is characterized by the presence of glycoside iridoids, flavonoids, phenylethanoids, and lignans in its chemical profile. In this work, we isolated aucubin and geniposide as major iridoids [3,9], verbascoside as a major phenylethanoid glycoside [5], and tenuifloroside, eudesmin, magnolin, kobusin, and sesamin as major lignans. Until now, tenuifloroside was the only furofuran lignan reported for *C. tenuiflora* [4], so this is the first time that eudesmin, magnolin, kobusin, and sesamin are reported for this species.

Isolated iridoids presented NF-κB/AP-1 inhibitory activity. Geniposide was more effective than aucubin as previously reported in a 12-O-Tetradecanoylphorbol-13-acetate (TPA)-induced mouse ear edema model [3]. Aucubin activity was due to the suppression of κB inhibitor (I κB) degradation and p65 subunit translocation [33] and geniposide by p38, Erk1/2, and JNK phosphorylation inhibition via AP-1 and NF-κB through the attenuation of IκB degradation in macrophages [34].

According to the EC_50_ results, methoxy and methylenedioxy groups were important in NF-κB/AP-1 inhibitory activity, which increased with the number of methoxy groups, as long as there were not methylenedioxy groups on the lignan structure. This result is similar to that found by Yang et al. [35] on RAW 264.7 macrophage cells, where they analyzed the role of methoxy groups on the anti-inflammatory effect of curcumin and realized that curcumin (Cur-OCH_3_) potently inhibited inflammation in vitro, suppressing lipopolysaccharide (LPS)-induced phosphorylation of IκB kinase (IKK) and degradation of IκBα, more than its synthetic analogues (Cur-OH, Cur-Br > Cur-H, Cur-F, Cur-CH3, Cur-Cl, > Cur-NO_2_).

Pharmacodynamic interactions were found in binary mixtures using verbascoside as a leader compound. Verbascoside was selected as a leader compound because it is a major constitutive PSM in wild *C. tenuiflora* methanolic extracts and has demonstrated NF-κB/AP-1 inhibitory activity [4,36,37,38]. Verbascoside-aucubin and verbascoside-kobusin mixtures showed synergism and verbascoside-tenuifloroside displayed a subadditivity effect in the inhibition of NF-κB/AP-1. The synergistic effect could be due to a simultaneous inhibitory activity between verbascoside and aucubin or kobusin on the different receptors of the NF-κB/AP-1 signaling pathway. Conversely, verbascoside inhibits TAK-1 phosphorylation through Src-homology 2 domain-containing protein tyrosine phosphatases (SHP-1) activation [26]. TAK-1 is the key regulatory point of the bifurcation to AP-1 or NF-κB pathway, and an inhibition upstream to NF-κB or AP-1 nucleus translocation is desirable, aiming to reduce proinflammatory cytokine signaling and inflammatory gene expression. Aucubin blocks nuclear translocation of the p65 subunit of NF-κB and suppresses degradation of the κB inhibitor α (IκBα) [39]. Kobusin also inhibits nuclear translocation of the p65 subunit of NF-κB and IκBα phosphorylation [40]. Also, sesamin, a furofuran lignan, inhibits the expression of TLR-4 [21,41], which is the LPS receptor that initiates the signaling cascade for the activation of NF-kB and AP-1. Kobusin is a furofuran lignan with similar structure to sesamin and it is expected to have a similar mechanism of action. The upstream inhibition of TLR-4 by lignans could be the reason why verbascoside-kobusin presented a higher inhibition ratio than verbascoside-aucubin.

**Figure 2:**
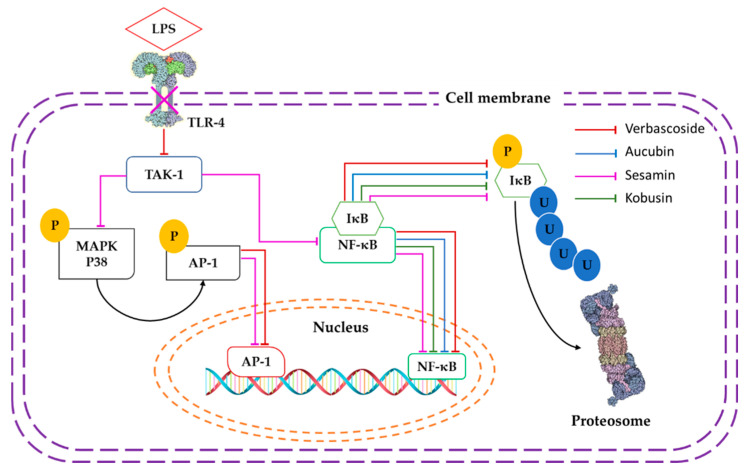
Proposed inhibition mechanism of the verbascoside-kobusin and verbascoside-aucubin mixtures in RAW-Blue ™ cells. Continuous line indicates enzyme inhibition. Crossed line indicates inhibition of expression. TLR-4: toll-like receptor 4. AP-1: activator protein. IκB: inhibitor of κB. TAK-1: transforming growth factor-β-activated kinase 1, MAPK p38: mitogen activated protein kinase p38. NF-κB: nuclear factor κB. U: ubiquitin, P: phosphate.

However, verbascoside-tenuifloroside binary mixture did not present results as expected; we thought that it was going to have the same behavior as the verbascoside-kobusin mixture (Figure 2), but only subadditivity was observed, possibly due to chemical interactions between verbascoside and tenuifloroside that might form an adduct with lower pharmacological effect. The adduct can be formed by sugar–sugar interactions between the glucopyranose moieties of the disaccharides in verbascoside and tenuifloroside through hydrogen bonds [42]; sugar–aromatic interactions [43,44] by the formation of CH/π bond between aromatic rings and the glucopyranose moieties presented in tenuifloroside and verbascoside; and/or hydrogen bonds between carbonyl and hydroxyl groups of both compounds.

Moreover, the experimental strategy using a leader compound with known pharmacological activity (NF-κB/AP-1 inhibition) allowed us to identify pharmacodynamic interactions that in a conventional bio-guided separation would not be possible. In bio-guided separation, verbascoside (middle polarity) and non-glycosylated lignans as kobusin (slightly polar) would not be in the same fraction because of their difference in polarity, in other words, following a traditional bio-guided separation would be unlikely to find the verbascoside-kobusin pharmacodynamic interaction.

## 4. Materials and Methods

### 4.1. Plant Material

Ten plants of wild *C. tenuiflora,* free of potential host plants in 40 cm of diameter, were collected from Parque Nacional Iztaccíhuatl-Popocatépetl (19°05′20″ N, 98°40′25.3″ W, 3446 m.a.s.l), State of Mexico, Mexico, in February 2016. *C. tenuiflora* plants were identified by MSc. Gabriel Flores Franco and, a voucher specimen (13234) was deposited at the Universidad Autónoma del Estado de Morelos herbarium.

### 4.2. Extraction and Isolation of Chemical Compounds from Methanolic Extracts

The aerial part was separated from the roots and it was air-dried under dark conditions. This plant material (920 g) was ground and extracted with methanol by maceration at room temperature for 48 h. The liquid extract was filtered (No. 1 Whatman filter paper) and concentrated to dryness at 40 °C under low pressure (Büchi-490 rotary evaporator). Solid extract (172 g) was stored at 4 °C for chromatographic separation.

The methanolic extract was subjected to bipartition with an immiscible mixture of ethyl acetate/water. The aqueous fraction (Aqf) was used to separate polar compounds and the ethyl acetate fraction (EAf) was extracted with middle and less polar compounds.

Aqf (2 g) was separated using a reverse phase silica gel column (LiChroprep ^®^ RP-18; 40–63 µm) and H_2_O-CH_3_CN solvent gradient system (Column 1), given 81 samples grouped in 25 fractions (C1F1-C1F25). According to thin layer chromatography (TLC), fraction 1 (C1F1), 8 (C1F8), and 20 (C1F20) contained isolated compounds and thus, were analyzed by high-performance liquid chromatography (HPLC). C1F1 (1, 45.8 mg) displayed a retention time (RT) of 4.14 min (Figure S1) and UV spectra λ_max_ 195 nm; C1F8 (2, 46.4 mg) was 8.33 min (Figure S2) and λ_max_ 238 nm, and C1F20 (3, 10 mg) was 10.34 min (Figure S4) and UV spectra λ_max_ 207, 229, and 282 nm.

EAf (6 g) was used for column 2 with the CH_2_Cl_2_-CH_3_OH solvent gradient system and column 3 with (CH_3_-CO_2_-C_2_H_5_)-C_6_H_6_, using normal phase silica gel 60 (Merck 0.063–0.200 mm). Column 2 was separated in 51 samples grouped in 19 fractions (C2F1-C2F19). C2F15 (4120 mg) had an RT of 9.33 min (Figure S3) and UV spectra λ_max_ 198, 219, and 330 nm. Column 3 displayed 81 samples grouped in 60 fractions (C3F1-C3F60). C3F29 (5, 42 mg), C3F50 (6, 90 mg), C3F52 (7, 28 mg), and C3F58 (8, 20 mg) seemed to be isolated compounds. C3F29 was 26.71 min (Figure S8) and λ_max_ 202, 236, and 286 nm; C3F50 was 25.67 (Figure S7) min and λ_max_ 207, 233, and 284 nm; C3F52 was 24.71 min (Figure S6) and λ_max_ 211 and 278 nm; and C3F58 23.28 min (Figure S5) and λ_max_ 208, 232, and 279 nm.

According to their UV spectra, compound 1 corresponded to a glycosidic iridoid without the carboxyl group at C4 (λ_max_:195 nm). The presence of this carboxyl group at C-4 in compound 2 was established by UV absorption data (λ_max_:238 nm). In the case of compound 3, this furofuran lignan produced the characteristic UV absorptions (λ_max_: 207, 229, and 282 nm) and *m*/*z* (ESI [M + Na]^+^: 674.26 *m*/*z*) of tenuifloroside previously described by Herrera-Ruiz et al. [4]. Compound 2 UV spectra and retention time matched with the geniposide reported by López-Rodríguez et al. [9]. The presence of geniposide was confirmed by mass spectra analysis (ESI [M + H]^+^: 389.52 *m*/*z*). Compound 4 was a phenylpropanoid glycoside, according to the *m*/*z* (ESI [M + H]^+^: 625.18 *m*/*z*). The RT and that reported by Gómez-Aguirre et al. [5] correspond to verbascoside. Compounds 5, 6, 7, and 8 were colorless needle-shaped crystals with an UV spectrum similar to tenuifloroside.

### 4.3. TLC and HPLC Analysis

Analytical TLC was carried out on a precoated Merck silica gel 60F_254_ or RP-18F_254_ plates. Komarowski reagent was used to derivatize terpenes and lignans and natural products reagent to visualize phenylethanoid glycosides.

HPLC separations were performed on a Waters 2695 separations module equipped with a Waters 2996 photodiode array detector and HPLC analysis was carried out using a LiChrospher^®^ 100 RP-18 column (4 mm × 250 mm, 5 µm) (Merck, Kenilworth, NJ, USA). The mobile phase consisted of two solvent reservoirs, A (H_2_O-Trifluoroacetic acid 0.05%) and B (CH_3_CN). The gradient system was as follows: 0–8 min, 100–0% B; 9–12 min, 90–10% B; 13–15 min, 80–20% B; 16–20 min, 70–30%, 21–25 min, 0–100% B, and 26–28 min 100–0% B. The flow rate was 1 mL/min and the injection volume was 10 µL [9]. Reservoir A was changed to H_2_O without TFA in the iridoids samples. The absorption was measured at λ = 205 nm to visualize iridoids without the carbonyl group at the C4 position, λ = 240 nm for iridoids with the carbonyl group in the C4 position, λ = 280 nm for furofuran lignans, λ = 330 nm for phenylethanoids, and λ = 360 nm for flavonoids.

### 4.4. UPLC Analysis

This chromatographic analysis was done using an Acquity UPLC (Waters, Milford MA, USA) provided by quaternary pump, autosampler column oven, and a photodiode array-detector. Chromatographic separations were performed in a UPLC BEH 1.7 m-C18 column at a flow rate of 0.4 mL/min. The mobile phase consisted of 0.1% formic acid in water (A) and 0.1% formic acid in acetonitrile (B). The column was held at 100% of A for 1 min and subsequently ramped to 100% of B (curve 6) over 11 min, followed by a 4 min period at 100% of B before a rapid return to 100% of A, and an equilibration period of 2 min. The column was maintained at temperatures of 40 °C. The injection volume was 5 µL and absorbance was measured at a range of wavelength from 190–600 nm. Mass spectrometry analysis was performed and analyzed in a triple quadrupole mass spectrometer (Waters) through an electrospray Z-spray ion source in ESI positive mode. Source and desolvation temperatures were 150 and 400 °C, respectively. A combination of cone voltage of 20 V and capillary voltage of 2.5 kV was used. Nitrogen was employed both as a desolvation gas and cone gas. An MS scan was performed using argon gas as the collision gas [45].

### 4.5. ^1^H- and ^13^C-NMR Experiments

One and two-dimensional Nuclear Magnetic Resonance (NMR) experiments (COSY, HSQC, HMBC) were performed on a Varian INOVA-400 instrument at 400 MHz CDCl_3_ or CD_3_OD were used as solvents with tetramethylsilane (TMS) as an internal standard. Chemical shifts (δ) are reported in ppm values and coupling constants are in Hz.

### 4.6. Raw-Blue™ Cells Culture Experiments

Raw-blue™ cells were cultured at 37 °C using manufacturer recommendations (InvivoGen) in a 24-well microplate until 70–80% confluence (1 × 10^5^ cells/well) was reached. This cell line expressed the secreted embryonic alkaline phosphatase (SEAP) gene under the control of a promoter inducible by the transcription factors NF-κB and AP-1. The different treatments were added to each well, after 1 h of incubation with lipopolysaccharide (LPS) at 1 µg/mL. The microplate was incubated for 24 h at 37 °C in a CO_2_ chamber at 5%. NF-κB and AP-1 activity was determined indirectly by quantifying SEAP activity in the supernatants. Fifty microliters of supernatant was collected in another 96-well microplate and 150 µL of the QUANTI-blue™ assay buffer (InvivoGen, San Diego, CA, USA) was added to measure the secreted alkaline phosphatase activity [46]. After one hour of incubation at 37 °C, absorbance was measured on a microplate reader (BIO-RAD iMark™) at 630 nm. Medium from cells without treatment was used as negative control. Cells + LPS were used as 100% of NF-κB/AP-1 activity. The inhibition activity (%) of NF-κB was calculated using the following equation:

(1) $$IA=\;\frac{A-B}{A}\;\times 100$$

where

*IA*: Inhibition activity (%),
*A*: Absorbance of 100% activity,
*B*: Absorbance of the treatment.

Indomethacin (1 µM) was used as a positive control for NF-κB/AP-1 inhibition. All experiments were performed four times with one repetition each.

#### 4.6.1. Cell Viability on Raw-Blue ™ Cells Using the MTT Method

Cell culture was performed according to specifications made in the previously section. Once a 70–80% confluence was reached, five concentrations in log_10_ of 100 µM (100, 10, 1, 0.1, and 0.01 µM) of all isolated compounds were evaluated. The different dilutions were prepared from a stock solution of 2000 µM from each compound. Compounds were diluted in ultrapure water using 0.02% tween 20. Ultrapure water with tween 20 (0.04%) was used as a control. The treated cells were incubated for 48 h at 37 °C in a humid atmosphere and 5% CO_2_. The test was done according to a modified method [47] by quadruplicate. After incubation, the medium was discarded, 80 µL of medium without fetal bovine serum and 20 µL of 3-[4,5-dimethylthiazol-2-yl]-2,5 diphenyl tetrazolium bromide (MTT) (5 mg/mL in phosphate buffered saline (PBS)) were added. Then, it was incubated for 24 h at 37 °C to allow formation of formazan crystals. The supernatant was removed and 100 μL isopropanol was added. Fifteen minutes of agitation were necessary to dissolve formazan crystals. The supernatant was taken and put in a new 96-well microplate. Absorbance was read in a microplate reader (Bio-rad iMark™) at 490 nm. The viability percentage was calculated by:

(2) $$\%\;Cell\;viabibility=\;\frac{C\;-\;T}{C\;}\;\times 100\;$$

where

*C*: Absorbance of the control,
*T*: Treatment absorbance.

#### 4.6.2. Inhibition of NF-κB/AP-1 by the Effective Concentration 50 (EC_50_) of Compounds Isolated from *C. tenuiflora* Using Raw-Blue™ Cells

To compare and obtain pharmacodynamic interactions in the binary mixtures, it was necessary to determine EC_50_ for each compound. Five concentrations in Log_10_ from 100 µM (100, 10, 1, 0.1, and 0.01 µM) of iridoids glycosides and lignans and, 200, 100, 10, 1, and 0.1 µM for verbascoside were evaluated in Raw-blue™ cells. Each compound was dissolved in ultrapure water with tween 20 at 0.02%. Inhibitory activity (%) of NF-κB/AP-1 was determined as explained in the Raw-blue™ cells culture experiments section. The EC_50_ was calculated by extrapolating the concentration in which 50% of the maximum effect was observed in a linearized effect-concentration model using the inverse.

#### 4.6.3. Inhibition of NF-κB/AP-1 in LPS Stimulated Raw-blue ™ Cells by Binary Mixtures of Iridoid Glycosides and Lignans with Verbascoside

Binary mixtures were prepared with verbascoside as leader and considering the EC_50_ of each compound. Treatments were 1.00AEC_50_/1.00BEC_50_, where A was verbascoside and B were aucubin, geniposide, sesamin, magnoline, kobusin, and tenuifloroside. Binary mixtures were prepared in a sterile conic tube, considering the EC_50_ of each compound as the final concentration in each well.

After incubating the Raw-blue™ cells in a 24-well microplate, each treatment was added and the inhibition activity (%) of NF-κB/AP-1 was calculated as described in the Raw-blue™ cells culture experiments section.

#### 4.6.4. Pharmacodynamic Interaction of Binary Mixtures of Iridoids and Lignans with Verbascoside in RAW Blue™ Cells

Isobolograms were performed to determine the type of pharmacodynamic interaction that presented the most active binary mixture of each group of compounds (lignans and iridoids glycoside with verbascoside). For this, the EC_50_ of the isolated compounds was taken into account, of which subsequent dilutions of the EC_50_ were evaluated in a fixed proportions scheme (1.00AEC_50_/0.00BEC_50_, 0.75AEC_50_/0.25EC_50_, 0.50AEC_50_/0.50BEC_50_, 0.25AEC_50_/0.75BEC_50_, 0.00AEC_50_/1.00BEC_50_, where A was verbascoside and B were aucubin, kobusin, or tenuifloroside). The inhibition ratio was calculated by:

(3) $$Inhibition\;ratio=\;\frac{OIA}{TIA\;}$$

where

*OIA*: Observed inhibition activity (%),
*TIA*: Expected inhibition activity (%).

Inhibition ratio close to 1 indicated additivity, <1 subadditivity, >1 synergism [48].

### 4.7. Statistical Analyses

EC_50_ was determined by linear regression. All experiments were performed in quadruplicate. Statistical analyses were carried out using SPSS software (IBM, version 25.0) using one-way ANOVA followed by Duncan’s multiple range test. *p*-values less than 0.05 were considered statistically significant.

## 5. Conclusions

All the compounds isolated from *C. tenuiflora* inhibited NF-κB/AP-1 on their own, magnolin was the most effective and aucubin was the least active. Methoxy groups are important for NF-κB/AP-1 inhibition activity of lignans. The inhibitory activity on NF-κB/AP-1 of the binary mixtures is explained by pharmacodynamic interactions; verbascoside-aucubin showed synergism when verbascoside concentration was higher than aucubin and subadditivity when the opposite. Verbascoside-kobusin mixture showed synergism evaluated, principally at 0.50EC_50_/0.50EC_50_ V/K. Verbascoside-tenuifloroside showed subadditivity in all concentrations, mainly 0.50EC_50_/0.50EC_50_ V/T. Verbascoside-kobusin could be a promising mixture to be used on diseases associated with increased NF-κB/AP-1 activity. The proposed experimental strategy using binary mixtures and a leader compound was effective in the search of pharmacodynamic interactions. These results are essential in the development of clinical treatments from *C. tenuiflora*. Also, this is the first time that non-glycosylated furofuran lignans were reported for this species, as well as the NF-κB/AP-1 inhibition activity for tenuifloroside and pharmacodynamic interactions between verbascoside-aucubin, verbascoside-kobusin, and verbascoside-tenuifloroside. Anti-inflammatory *C. tenuiflora*-based phytomedicines formulas must consider verbascoside iridoids and verbascoside lignans interactions. This information also led to aim the biotechnological approach in order to obtain *C. tenuiflora* plantlets or cells in bioreactors that biosynthetize verbascoside and kobusin, avoiding tenuifloroside production.

## Figures and Tables

**Table 1:** Parameters calculated from the concentration response curve, measured as inhibition of NF-kB/AP-1 activity, in the RAW-blue™ cell culture. E_max_ and EC_50_ of verbascoside, iridoids glycosides, and lignans isolated from *C. tenuiflora* methanolic extract.

Compound	Emax (% Inhibition)	EC_50_ (µM)
Verbascoside	55.55	10.08
Aucubin	40.00	0.08
Geniposide	57.47	0.01
Tenuifloroside	52.91	0.11
Magnolin	60.97	3.05
Eudesmin	56.82	0.006
Kobusin	45.45	2.37
Sesamin	52.63	0.01

**Table 2:** NF-κB/AP-1 inhibitory activity on RAW-Blue™ cells of binary mixtures using verbascoside as a leader compound.

Mixture	Expected NF-κB/AP-1 Inhibition (%)	Observed NF-κB/AP-1 Inhibition (%)	Inhibition Ratio
Indomethacin		36.31 ± 9.15 ^a^	
Verbascoside-aucubin	53.65	46.16 ± 3.67 ^a,b^	0.84
Verbascoside-geniposide	48.58	43.04 ± 5.59 ^a,b^	0.89
Verbascoside-tenuifloroside	60.18	37.99 ± 7.70 ^a^	0.63
Verbascoside-magnolin	52.95	39.33 ± 7.59 ^a,b^	0.74
Verbascoside-eudesmin	52.70	51.01 ± 7.99 ^b^	0.97
Verbascoside-kobusin	55.60	63.06 ± 5.90 ^b,c^	1.13
Verbascoside-sesamin	44.94	47.40 ± 16.28 ^b,c^	1.05

Same letters indicate no significant difference between groups. Duncan α < 0.05. Inhibition ratio close to 1 indicate additivity, <1 subadditivity, >1 synergism.

**Table 3:** Fixed proportions scheme of verbascoside-aucubin, verbascoside-kobusin, and verbascoside-tenuifloroside binary mixtures.

Binary Mixture	Expected NF-κB/AP-1 Inhibition (%)	Observed NF-κB/AP-1 Inhibition (%)	Inhibition Ratio
V-A proportion Indomethacin		33.22 ± 6.53 ^a^	
1.00/0:00	26.54	26.10 ± 4.24 ^a^	1.05
0.75/0.25	27.53	34.82 ± 8.37 ^a^	1.32
0.50/0.50	28.52	34.21 ± 4.15 ^a^	1.21
0.25/0.75	29.52	25.47 ± 10.65 ^a^	0.86
0.00/1.00	29.84	30.42 ± 4.20 ^a^	1.02
V-K proportion			
Indomethacin		34.07 ± 9.13 ^b,c^	
1.00/0:00	26.54	22.77 ± 5.57 ^a^^,b^	0.91
0.75/0.25	27.46	33.93 ± 4.07 ^b,c^	1.24
0.50/0.50	28.38	43.23 ± 5.17 ^c^	1.56
0.25/0.75	29.29	36.52 ± 4.60 ^b,c^	1.26
0.00/1.00	30.21	29.10 ± 6.63 ^a^	0.95
V-T proportion			
Indomethacin		30.77 ± 4.91 ^b,c^	
1.00/0:00	26.54	26.45 ± 2.47 ^b^	0.93
0.75/0.25	28.60	20.59 ± 2.70 ª	0.72
0.50/0.50	30.66	14.16 ± 6.07 ª	0.46
0.25/0.75	32.72	28.66 ± 2.82 ^b^	0.91
0.00/1.00	34.78	37.98 ± 5.97 ^c^	1.07

V-A: verbascoside-aucubin, V-K: verbascoside-kobusin, V-T: verbasoside-tenuifloroside. Same letters indicate no significant difference between groups. Duncan α < 0.05. Inhibition ratio close to 1 indicate additivity, <1—subadditivity, >1—synergism.

## Data Availability

The data presented in this study are available within the article or supplementary material.

## Supplementary Materials

The following are available online, Table S1: Calculated and corrob-orated NF-κB/AP-1 inhibition activity at EC50 of isolated compounds from C. tenuiflora meth-anolic extract of aerial parts. Table S2: EC50 linearized equations of isolated compounds from C. tenuiflora methanolic extract of aerial parts. Figures S1–S8: Chromatogram. Figures S9–S16: Mass spectra. Figure S17: Inhibitory effect on NF-κB/AP-1 activation in RAW-Blue ™ cells from ver-bascoside-iridoid binary mixtures. V: verbascoside, A: aucubin, G: geniposide, V + A: verbasco-side-aucubin, V + G: verbascoside-geniposide. Figure S18: Inhibitory effect on the activation of NF- κB/AP-1 in RAW-Blue ™ cells from the verbascoside-lignan mixtures. V: verbascoside, T: tenuifloroside, M: magnolin, E: eudesmin, K: kobusin, S: sesamin, V + T: verbasco-side-tenuifloroside, V + M: verbascoside-magnolin, V + E: verbascoside-eudesmin, V + K: verbas-coside-kobusin, V + S: verbascoside-sesamin. Figures S19–S28: NMR spectrum. Figure S29: Effect of aucubin, geniposide, verbascoside, tenuifloroside, sesamin, eudesmin, kobusin, and magnolin from C. tenuiflora on Raw-blue™ cells viability.

## Sample Availability

Samples of the compounds are available from the authors.

## References

[1] Martínez M. (1994). Catálogo de Nombres Vulgares y Científicos de Plantas Mexicanas.

[2] Alonso-Castro A.J., Villarreal M.L., Salazar-Olivo L.A., Gomez-Sánchez M., Dominguez F., Garcia-Carranca A. (2011). Mexican medicinal plants used for cancer treatment: Pharmacological, phytochemical and ethnobotanical studies. J. Ethnopharmacol..

[3] Carrillo-Ocampo D., Bazaldúa-Gómez S., Bonilla-Barbosa J.R., Aburto-Amar R., Rodríguez-López V. (2013). Anti-inflammatory activity of iridoids and verbascoside isolated from *Castilleja tenuiflora*. Molecules.

[4] Herrera-Ruiz M., López-Rodríguez R., Trejo-Tapia G., Dominguez-Mendoza B.E., González-Cortazar M., Tortoriello J., Zamilpa A. (2015). A new furofuran lignan diglycoside and other secondary metabolites from the antidepressant extract of *Castilleja tenuiflora* Benth. Molecules.

[5] Gómez-Aguirre Y.A., Zamilpa A., González-Cortazar M., Trejo-Tapia G. (2012). Adventitious root cultures of *Castilleja tenuiflora* Benth. as a source of phenylethanoid glycosides. Ind. Crops Prod..

[6] Moreno-Escobar J.A., Bazaldúa S., Villarreal M.L., Bonilla-Barbosa J.R., Mendoza S., Rodríguez-López V. (2011). Cytotoxic and antioxidant activities of selected Lamiales species from Mexico. Pharm. Biol..

[7] López-Laredo A., Gómez-Aguirre Y., Medina-Pérez V., Salcedo-Morales G., Sepúlveda-Jiménez G., Trejo-Tapia G. (2012). Variation in antioxidant properties and phenolics concentration in different organs of wild growing and greenhouse cultivated *Castilleja tenuiflora* Benth. Acta Physiol. Plant..

[8] Sanchez P.M., Villarreal M.L., Herrera-Ruiz M., Zamilpa A., Jiménez-Ferrer E., Trejo-Tapia G. (2013). In vivo anti-inflammatory and anti-ulcerogenic activities of extracts from wild growing and in vitro plants of *Castilleja tenuiflora* Benth. (Orobanchaceae). J. Ethnopharmacol..

[9] López-Rodríguez R., Herrera-Ruiz M., Trejo-Tapia G., Domínguez-Mendoza B.E., González-Cortazar M., Zamilpa A. (2019). In vivo gastroprotective and antidepressant effects of iridoids, verbascoside and tenuifloroside from *Castilleja tenuiflora* Benth. Molecules.

[10] Seyfi D., Behzad S.B., Nabiuni M., Parivar K., Tahmaseb M., Amini E. (2018). Verbascoside attenuates Rac-1 and HIF-1α signaling cascade in colorectal cancer cells. Med. Chem..

[11] Kartini S.P., Piyaviriyakul S., Thongpraditchote S., Siripong P., Vallisuta O. (2017). Effects of plantago major extracts and its chemical compounds on proliferation of cancer cells and cytokines production of lipopolysaccharide-activated THP-1 macrophages. Pharmacogn. Mag..

[12] Niu J., Straubinger R.M., Mager D.E. (2019). Pharmacodynamic Drug-Drug Interactions. Clin. Pharmacol. Ther..

[13] Cascorbi I. (2012). Drug interactions—Principles, examples and clinical consequences. Deutsches Arzteblatt Int..

[14] Einbond L.S., Shimizu M., Ma H., Wu H., Goldsberry S., Sicular S., Panjikaran M., Genovese G., Cruz E. (2008). Actein inhibits the Na+-K+-ATPase and enhances the growth inhibitory effect of digitoxin on human breast cancer cells. Biochem. Biophys. Res. Commun..

[15] Attia Y.M., El-Kersh D.M., Wagdy H.A., Elmazar M.M. (2018). Verbascoside: Identification, quantification, and potential sensitization of colorectal cancer cells to 5-FU by targeting PI3K/AKT pathway. Sci. Rep..

[16] Luszczki J.J., Ratnaraj N., Patsalos P.N., Czuczwar S.J. (2005). Pharmacodynamic and/or pharmacokinetic characteristics of interactions between loreclezole and four conventional antiepileptic drugs in pentylenetetrazole-induced seizures in mice: An isobolographic analysis. Epilepsy Behav..

[17] Greten F.R., Grivennikov S.I. (2019). Inflammation and Cancer: Triggers, Mechanisms, and Consequences. Immunity.

[18] Kinney J.W., Bemiller S.M., Murtishaw A.S., Leisgang A.M., Salazar A.M., Lamb B.T. (2018). Inflammation as a central mechanism in Alzheimer’s disease. Alzheimers Dement..

[19] Bäck M., Yurdagul A., Tabas I., Öörni K., Kovanen P.T. (2019). Inflammation and its resolution in atherosclerosis: Mediators and therapeutic opportunities. Nat. Rev. Cardiol..

[20] Quinton L.J., Walkey A.J., Mizgerd J.P. (2018). Integrative physiology of pneumonia. Physiol. Rev..

[21] Guo Q., Wang Y., Xu D., Nossent J., Pavlos N.J., Xu J. (2018). Rheumatoid arthritis: Pathological mechanisms and modern pharmacologic therapies. J. Bone Res..

[22] Tsalamandris S., Antonopoulos A.S., Oikonomou E., Papamikroulis G.A., Vogiatzi G., Papaioannou S., Deftereos S., Tousoulis D. (2019). The role of inflammation in diabetes: Current concepts and future perspectives. Eur. Cardiol..

[23] Chen L., Deng H., Cui H., Fang J., Zuo Z., Deng J., Li Y., Wang X., Zhao L. (2017). Inflammatory responses and inflammation-associated diseases in organs. Oncotarget.

[24] Mu H.X., Lin C.Y., Huang L.F., Yang D.J., Lu A.P., Han Q.B., Bian Z.X. (2016). A novel coumarin, (+)-3′-angeloxyloxy-4′-keto-3′,4′-dihydroseselin, isolated from *Bupleurum malconense* (Chaihu) inhibited NF-κB activity. Chin. Med..

[25] Iitsuka H., Koizumi K., Suzaki M., Otsuka Y., Jo M., Shibahara N. (2020). Immunostimulatory effects of cell wall-based nanoparticles in boiled *Glycyrrhizae* radix water extracts involves TLR4. Biomed. Rep..

[26] Pesce M., Franceschelli S., Ferrone A., De Lutiis M.A., Patruno A., Grilli A., Felaco M., Speranza L. (2015). Verbascoside down-regulates some pro-inflammatory signal transduction pathways by increasing the activity of tyrosine phosphatase SHP-1 in the U937 cell line. J. Mol. Cell Med..

[27] Ramírez-Cisneros M.A., Rios M., Aguilar-Guadarrama A., Rao P., Aburto-Amar R., Rodríguez-López V. (2015). In vitro COX-1 and COX-2 enzyme inhibitory activities of iridoids from *Penstemon barbatus*, *Castilleja tenuiflora*, *Cresentia alata* and *Vitex molli*. Bioorg. Med. Chem. Lett..

[28] Ersoz T., Berkman M.Z., Tasdemir D., Ireland C.M., Calis I. (2000). An iridoid glucoside from *Euphrasia pectinata*. J. Nat. Prod..

[29] Calle J. (2007). Aislamiento, purificación e identificación de sesamina a partir de lodos de microfiltrado en la fabricación del aceite virgen de *Sesamum indicum* L. (ajonjolí). Rev. Colomb. Cienc. Quím. Farm..

[30] Chang W., Kim K., Lee I.K., Choi S., Lee K. (2009). Phytochemical constituents of *Geranum eriostemon*. Nat. Prod. Sci..

[31] Hao L., Zhi S., Da-Guang L., Tian-Yi Z., Feng L., Kai Z., Kui L., Liang Y., Jing H., Jian-Ping L. (2015). Anticonvulsant and sedative effects of eudesmin isolated from *Acorus tatarinowii* on mice and rats. Phytother. Res..

[32] Miyazawa M., Ishikawa Y., Kasahara H., Yamanaka J., Kameoka H. (1994). An insect growth inhibitory lignan from flower buds of *Magnolia fargesii*. Phytochemistry.

[33] Jain H., Dhingra N., Narsinghani T., Sharma R. (2016). Insights into the mechanism of natural terpenoids as NF-κB inhibitors: An overview on their anticancer potential. Exp. Oncol..

[34] Shi Q., Cao J., Fang L., Zhao H., Liu Z., Ran J., Zheng X., Li X., Zhou Y., Ge D. (2014). Geniposide suppresses LPS-induced nitric oxide, PGE2 and inflammatory cytokine by downregulating NF-κB, MAPK and AP-1 signaling pathways in macrophages. Int. Immunopharmacol..

[35] Yang H., Du Z., Wang W., Song M., Sanidad K., Sukamtoh E., Zheng J., Tian L., Xiao H., Liu Z. (2017). Structure-activity relationship of curcumin: Role of the methoxy group in anti-inflammatory and anticolitis effects of curcumin. J. Agric. Food Chem..

[36] Lee S.Y., Lee K.S., Yi S.H., Kook S.H., Lee J.C. (2013). Acteoside suppresses RANKL-mediated osteoclastogenesis by inhibiting c-Fos induction and NF-κB pathway and attenuating ROS production. PLoS ONE.

[37] Sipahi H., Gostner J.M., Becker K., Charehsaz M., Kirmizibekmez H., Schennach H., Aydin A., Fuchs D. (2016). Bioactivites of two common polyphenolic compounds: Verbascoside and catechin. Pharm. Biol..

[38] Khullar M., Sharma A., Wani A., Sharma N., Sharma N., Chandan B.K., Kumar A., Ahmed Z. (2019). Acteoside ameliorates inflammatory responses through NF-kB pathway in alcohol induced hepatic damage. Int. Immunopharmacol..

[39] Jeong H.J., Koo H.N., Na H.J., Kim M.S., Hong S.H., Eom J.W., Kim K.S., Shin T.Y., Kim H.M. (2002). Inhibition of TNF-α and Il-6 production by aucubin through blockade of NF-κB activation in RBL-2H3 mast cells. Cytokine.

[40] Kim J.Y., Lim H.J., Lee D.Y., Kim J.S., Kim D.H., Lee H.J., Kim H.D., Jeon R., Ryu J.H. (2009). In vitro anti-inflammatory activity of lignans isolated from *Magnolia fargesii*. Bioorg. Med. Chem. Lett..

[41] Udomruk S., Kaewmool C., Pothacharoen P., Phitak T., Kongtawelert P. (2018). Sesamin suppresses LPS-induced microglial activation via regulation of TLR4 expression. J. Funct. Food.

[42] Mason P.E., Neilson G.W., Enderby J.E., Saboungi M.L., Brady J.W. (2005). Structure of aqueous glucose solutions as determined by neutron diffraction with isotopic substitution experiments and molecular dynamics calculations. J. Phys. Chem. B.

[43] Asensio J.L., Ardá A., Cañada F.J., Jiménez-Barbero J. (2013). Carbohydrate—Aromatic interactions. Acc. Chem. Res..

[44] Spiwok V. (2017). CH/π Interactions in Carbohydrate Recognition. Molecules.

[45] Méndez-Martínez M., Trejo-Moreno C., Maldonado-Mejía L., Ezquivel-Guadarrama F., Pedraza-Chaverri J., Zamilpa A., Medina-Campos O., Alarcón-Aguilar F., Almanza-Pérez J., Contreras-Nuñez E. (2019). Effect of *Cucumis sativus* on dysfunctional 3T3-L1 adipocytes. Sci. Rep..

[46] Medrano-Jiménez E., Jiménez-Ferrer I., Pedraza-Escalona M., Ramírez-Serrano C., Álvarez-Arellano L., Cortés-Mendoza J., Herrera-Ruiz M., Jiménez-Ferrer E., Zamilpa A., Tortoriello J. (2019). *Malva parviflora* extract ameliorates the deleterious effects of a high fat diet on the cognitive deficit in a mouse model of Alzheimer’s disease by restoring microglial function via a PPAR-γ-dependent mechanism. J. Neuroinflamm..

[47] Mosmann T. (1983). Rapid colorimetric assay for cellular growth and survival: Application to proliferation and cytotoxicity assays. J. Immunol. Methods.

[48] Frum Y., Viljoen A.M., Van Heerden F.R. (2007). Verbascoside and luteolin-5-O-β-D-glucoside isolated from *Halleria lucida* L. exhibit antagonistic anti-oxidant properties in vitro. S. Afr. J. Bot..

