# Supplementary material for: Synergism and Subadditivity of Verbascoside-Lignans and -Iridoids Binary Mixtures Isolated from Castilleja tenuiflora Benth. on NF-κB/AP-1 Inhibition Activity
Source: Molecules. 2021 Jan 21;26(3):547. doi: 10.3390/molecules26030547 (PMC7865359; doi:10.3390/molecules26030547)
Supplement: Supplementary file 1 [file molecules-26-00547-s001.pdf]

molecules

Supplementary material

Article

Synergism and subadditivity of verbascoside-lignans and -iridoids binary mixtures isolated from *Castilleja tenuiflora* Benth. on the NF-κB/AP-1 inhibition activity

Luis David Arango-De la Pava[1], Alejandro Zamilpa[2,*], José Luis Trejo-Espino[1], Blanca Eda Domínguez-Mendoza[3], Enrique Jiménez-Ferrer[2], Leonor Pérez-Martínez[4] and Gabriela Trejo-Tapia[1,*]

[1] Instituto Politécnico Nacional, Centro de Desarrollo de Productos Bióticos, Yautepec, Morelos, 62731, Mexico; larangod1300@alumno.ipn.mx (L.D.A.-D); jtrejo@ipn.mx (J.L.T.-E);
[2] Instituto Mexicano del Seguro Social, Centro de Investigación Biomédica del Sur, Xochitepec, Morelos, 62790, Mexico; enriqueferrer_mx@yahoo.com (E.J.-F)
[3] Universidad Autónoma del Estado de Morelos, Centro de Investigaciones Químicas, Cuernavaca, Morelos 62209 México; bed@uaem.mx (B.E.D.-M)
[4] Universidad Nacional Autónoma de México, Instituto de Biotecnología, Cuernavaca, Morelos 62210 México; leonor@ibt.unam.mx (L.P.-M)
* Correspondence: gttapia@ipn.mx (G.T.-T.); azamilpa_2000@yahoo.com.mx (A.Z.)

molecules

Table of contents:

- **Figure S1**. Chromatogram of aucubin at 205 nm
- **Figure S2.** Chromatogram of geniposide at 240 nm
- **Figure S3.** Chromatogram of verbascoside at 330 nm
- **Figure S4.** Chromatogram of tenuifloroside at 280 nm
- **Figure S5.** Chromatogram of magnolin at 280 nm
- **Figure S6**. Chromatogram of eudesmin at 280 nm
- **Figure S7.** Chromatogram of kobusin at 280 nm
- **Figure S8**. Chromatogram of sesamin at 280 nm
- **Figure S9.** Mass spectra of aucubin
- **Figure S10.** Mass spectra of eudesmin
- **Figure S11.** Mass spectra of geniposide
- **Figure S12.** Mass spectra of tenuifloroside
- **Figure S13.** Mass spectra of verbascoside
- **Figure S14.** Mass spectra of magnolin
- **Figure S15.** Mass spectra of kobusin
- **Figure S16.** Mass spectra of sesamin
- **Figure S17.** Inhibitory effect on NF-κB/AP-1 activation in RAW-Blue ™ cells from verbascoside-iridoid binary mixtures. V: verbascoside, A: aucubin, G: geniposide, V + A: verbascoside-aucubin, V + G: verbascoside-geniposide
- **Figure S18.** Inhibitory effect on the activation of NF- κB/AP-1 in RAW-Blue ™ cells from the verbascoside-lignan mixtures. V: verbascoside, T: tenuifloroside, M: magnolin, E: eudesmin, K: kobusin, S: sesamin, V + T: verbascoside-tenuifloroside, V + M: verbascoside-magnolin, V + E: verbascoside-eudesmin, V + K: verbascoside-kobusin, V + S: verbascoside-sesamin
- **Figure S19**. ^{1}H-NMR spectrum of aucubin
- **Figure S20.** ^{13}C-NMR spectrum of aucubin

- **Figure S21.** ^{1}H-NMR spectrum of sesamin
- **Figure S22.** ^{13}C-NMR spectrum of sesamin
- **Figure S23.** ^{1}H-NMR spectrum of kobusin
- **Figure S24.** ^{13}C-NMR spectrum of kobusin
- **Figure S25.** ^{1}H-NMR spectrum of eudesmin
- **Figure S26.** ^{13}C-NMR spectrum of eudesmin
- **Figure S27**. ^{1}H-NMR spectrum of magnolin
- **Figure S28.** ^{13}C-NMR spectrum of magnolin
- **Figure S29.** Effect of aucubin, geniposide, verbascoside, tenuifloroside, sesamin, eudesmin, kobusin, and magnolin from *C. tenuiflora* on Raw-blue™ cells viability.
- **Table S1**. Calculated and corroborated NF-κB/AP-1 inhibition activity at EC_{50} of isolated compounds from *C. tenuiflora* methanolic extract of aerial parts.
- **Table S2**. EC_{50} linearized equations of isolated compounds from *C. tenuiflora* methanolic extract of aerial parts

molecules

MDPI

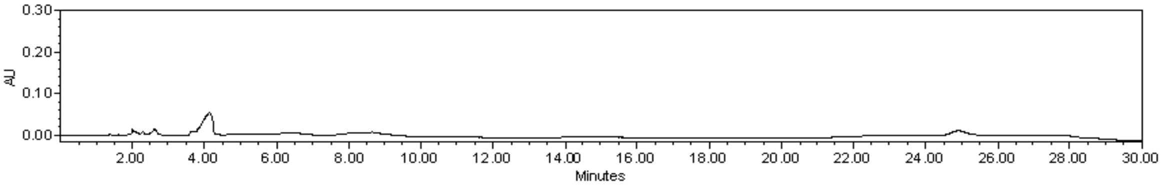

Figure S1. Chromatogram of aucubin at 205 nm

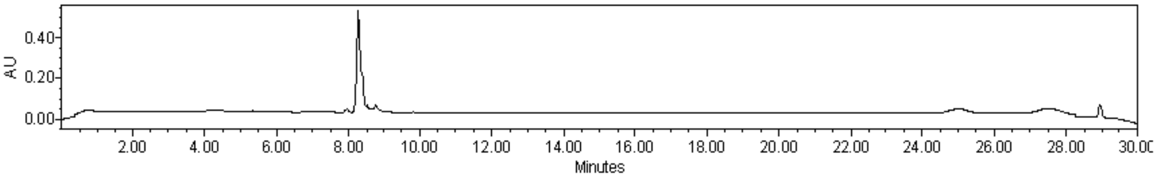

Figure S2. Chromatogram of geniposide at 240 nm

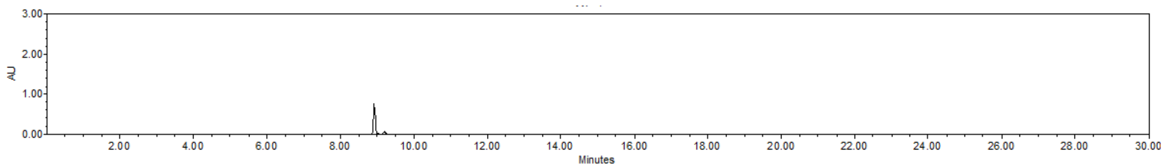

Figure S3. Chromatogram of verbascoside at 330 nm

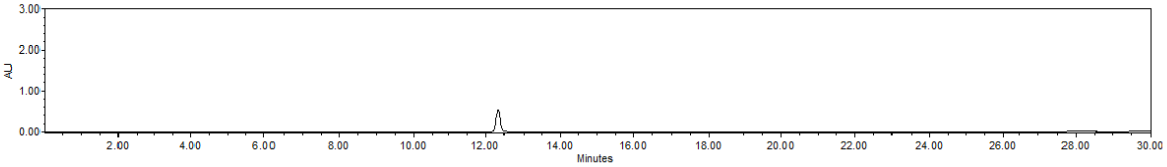

Figure S4. Chromatogram of tenuifloroside at 280 nm

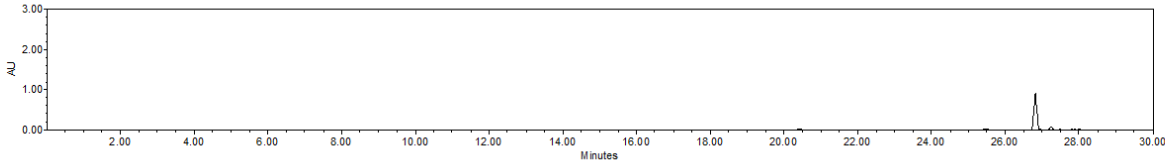

Figure S5. Chromatogram of magnolin at 280 nm

molecules

MDPI

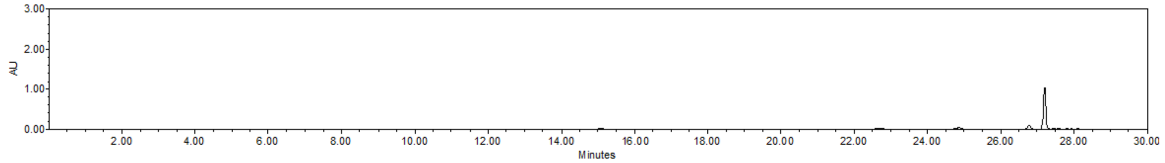

Figure S6. Chromatogram of eudesmin at 280 nm

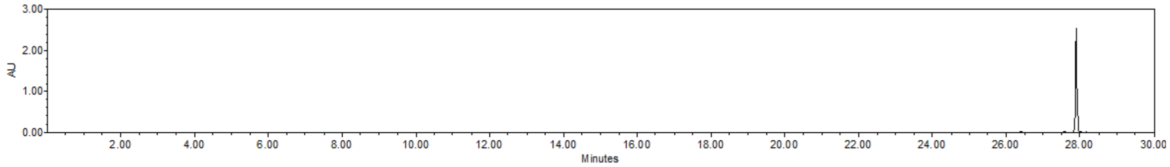

Figure S7. Chromatogram of kobusin at 280 nm

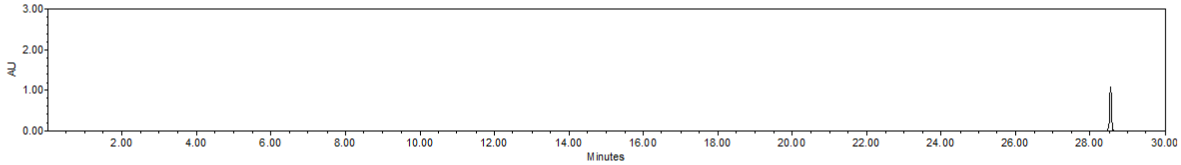

Figure S8. Chromatogram of sesamin at 280 nm

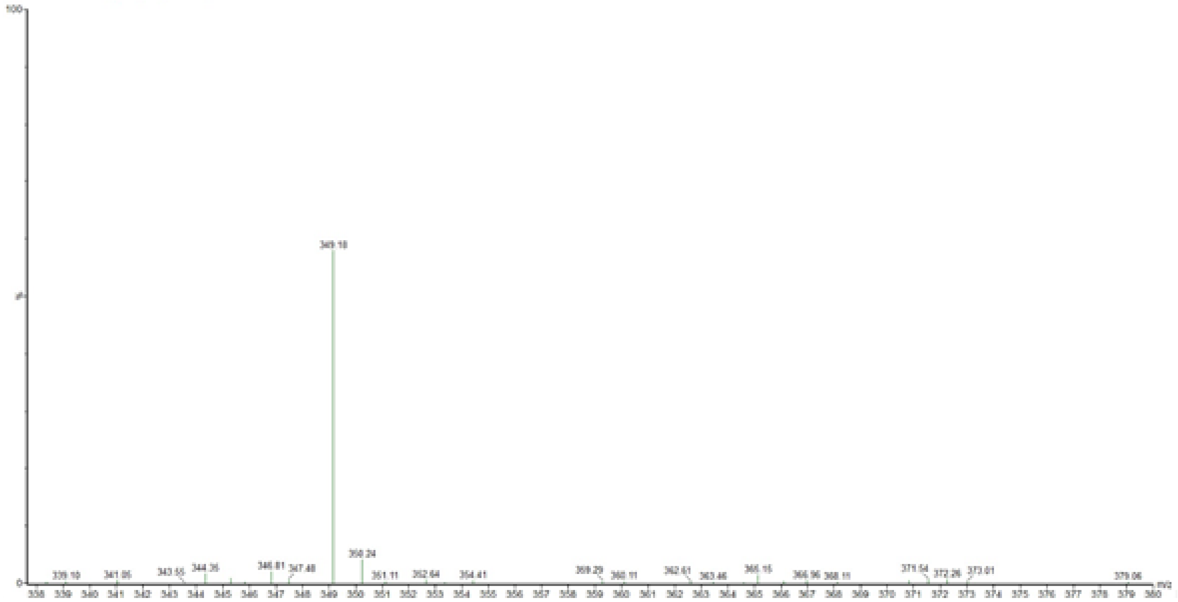

Figure S9. Mass spectra of aucubin

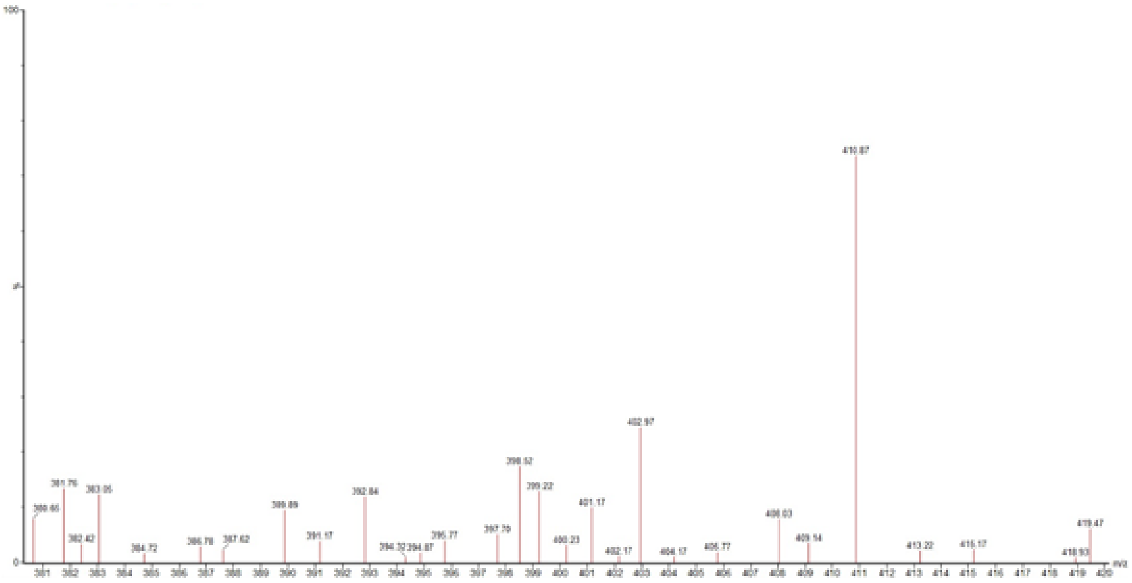

Figure S10. Mass spectra of eudesmin

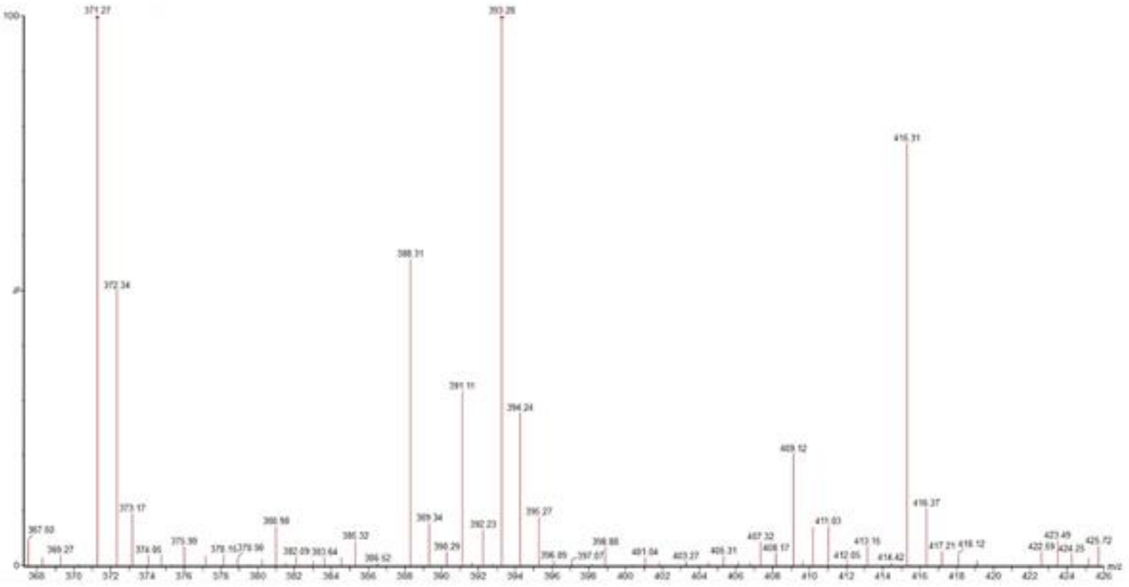

Figure S11. Mass spectra of geniposide

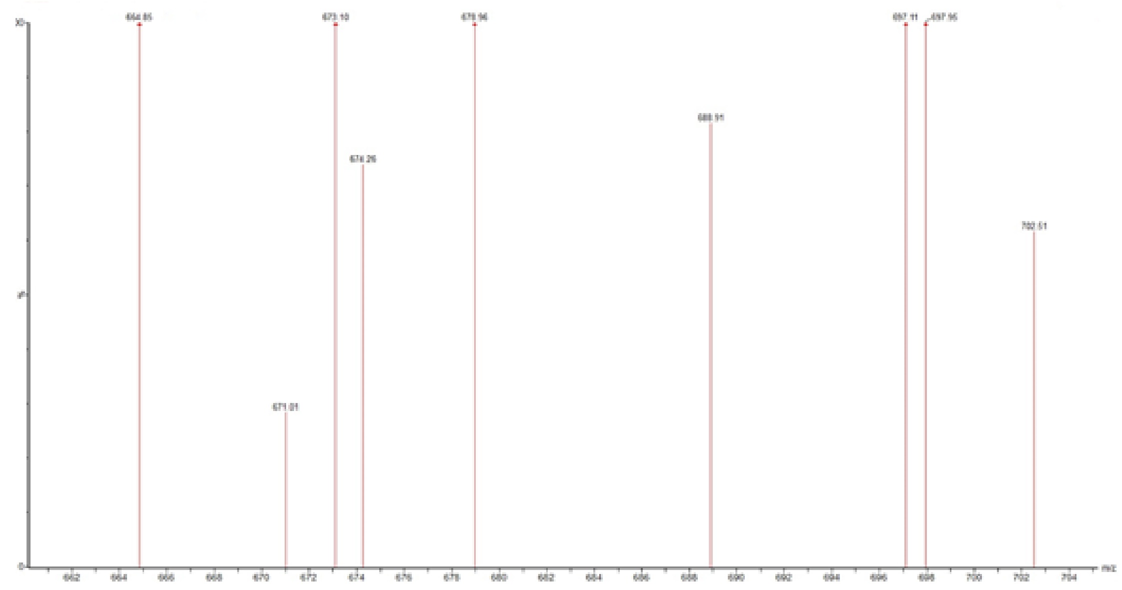

Figure S12. Mass spectra of tenuifloroside

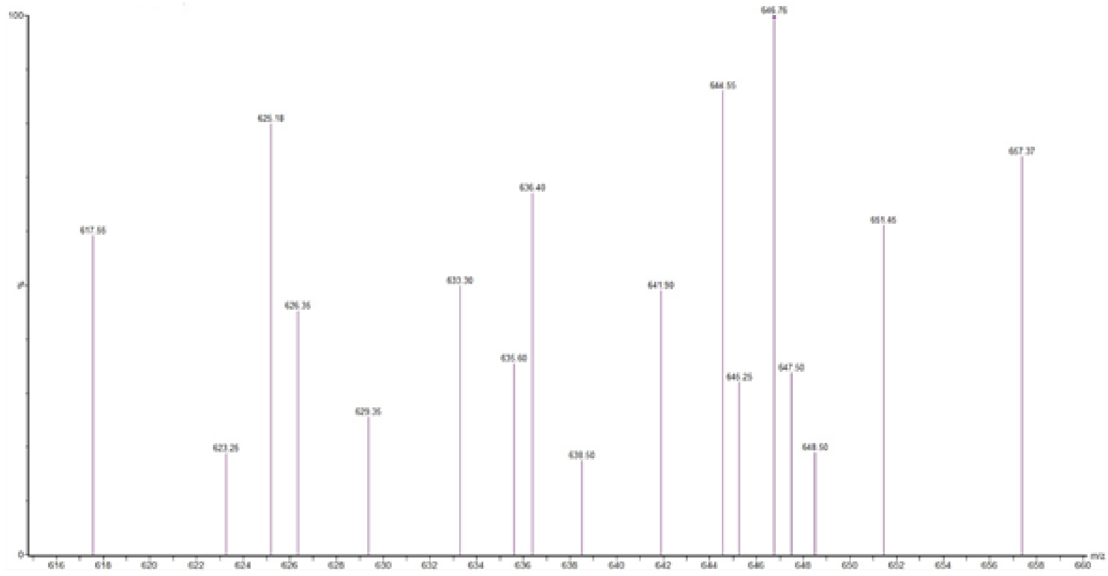

Figure S13. Mass spectra of verbascoside

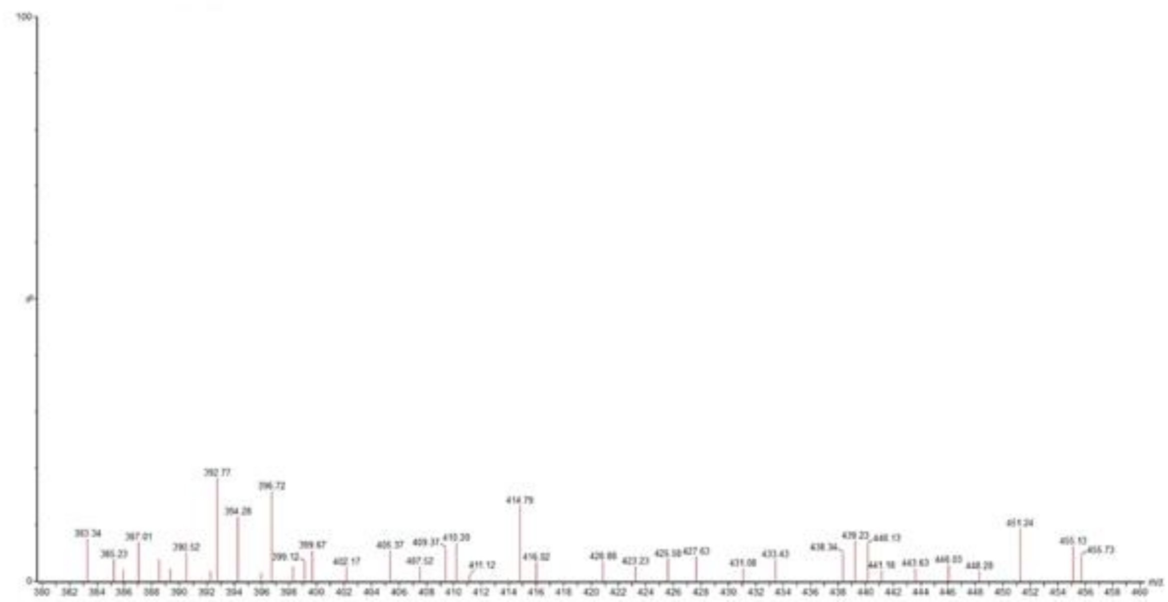

Figure S14. Mass spectra of magnolin

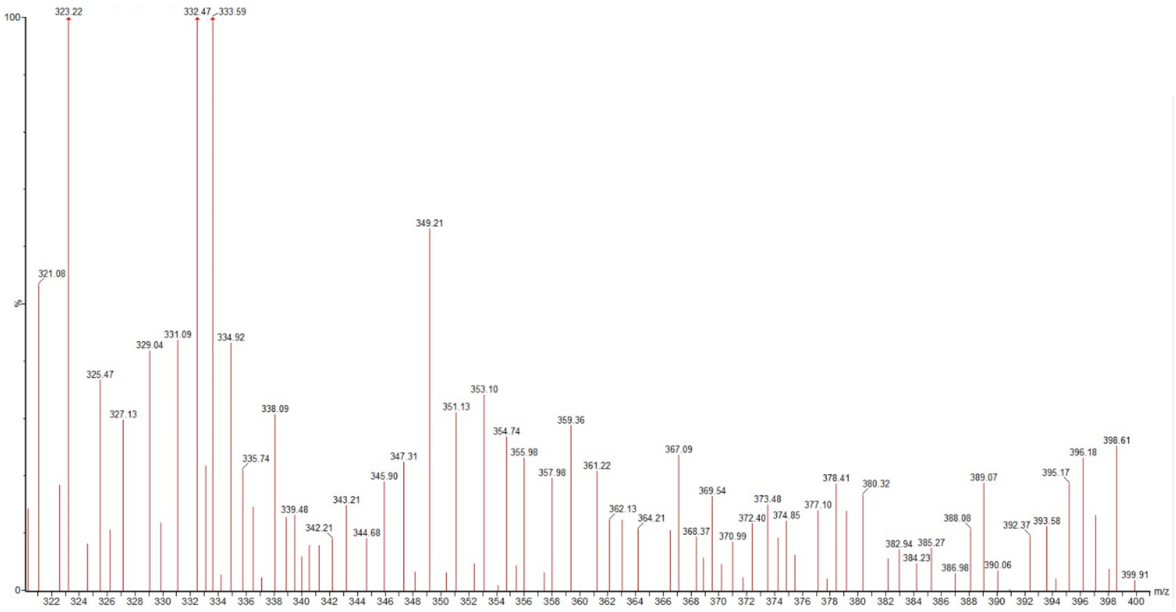

Figure S15. Mass spectra of kobusin

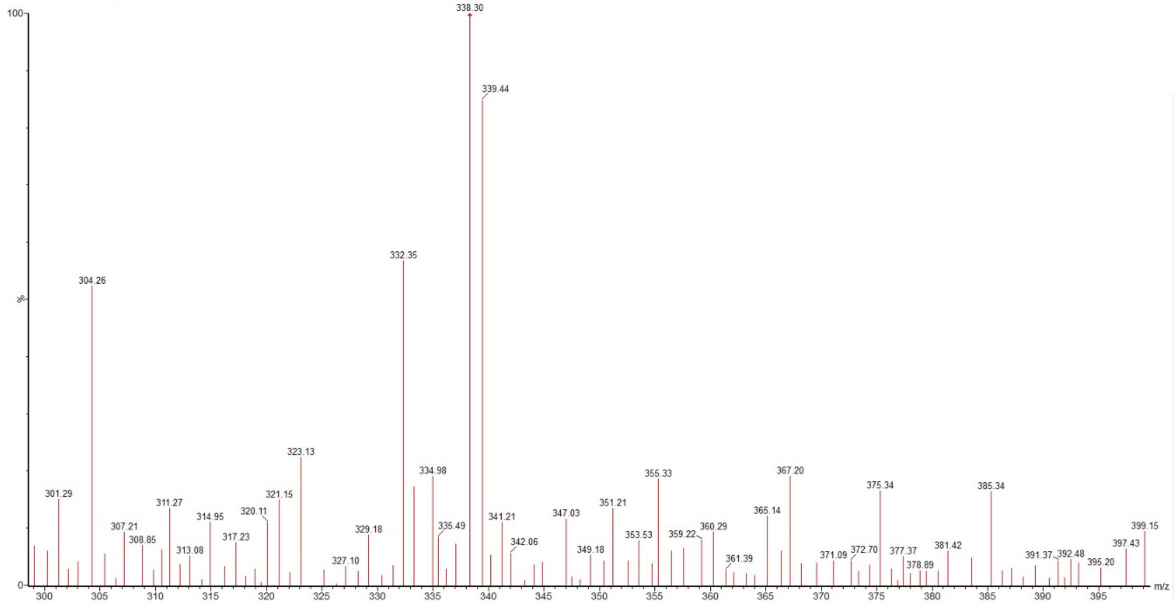

Figure S16. Mass spectra of sesamin

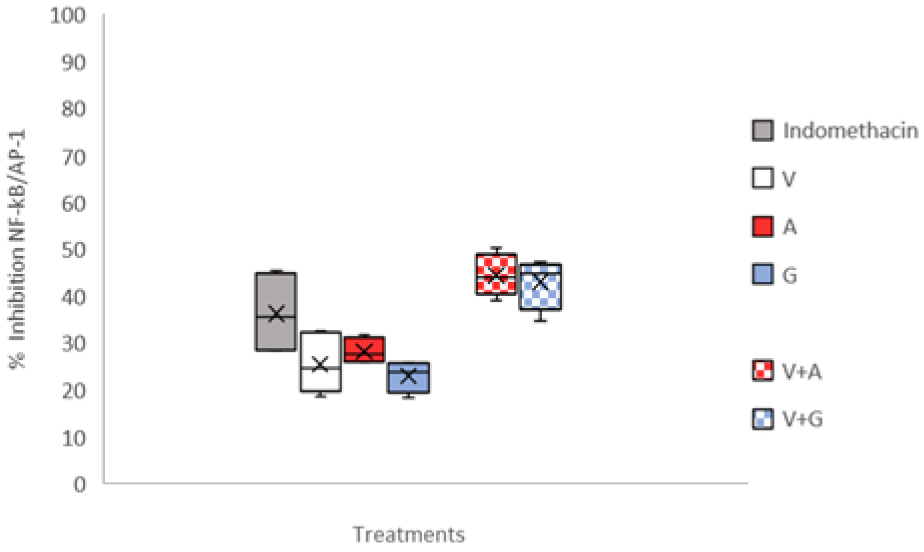

Figure S17. Inhibitory effect on NF-κB/AP-1 activation in RAW-Blue ™ cells from verbascoide-iridoid binary mixtures. V: verbascoside, A: aucubin, G: geniposide, V + A: verbascoside-aucubin, V + G: verbascoside-geniposide

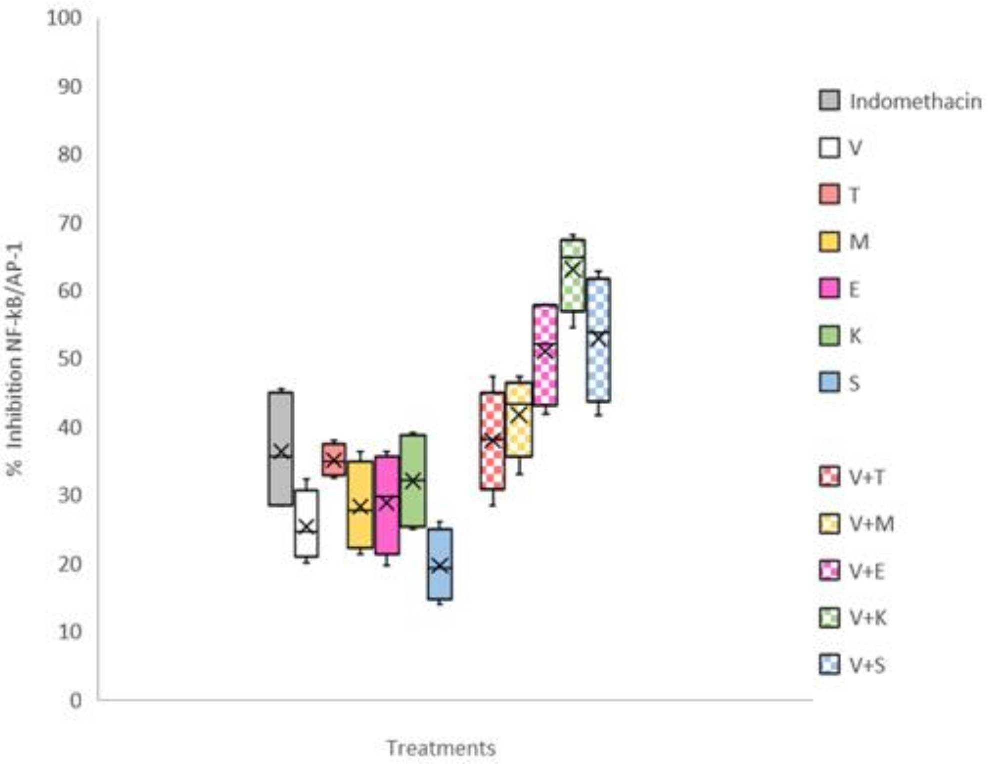

Figure S18. Inhibitory effect on the activation of NF- κB/AP-1 in RAW-Blue ™ cells from the verbascoside-lignan binary mixtures. V: verbascoside, T: tenuifloroside, M: magnolin, E: eudesmin, K: kobusin, S: sesamin, V + T: verbascoside-tenuifloroside, V + M: verbascoside-magnolin, V + E: verbascoside-eudesmin, V + K: verbascoside-kobusin, V + S: verbascoside-sesamin

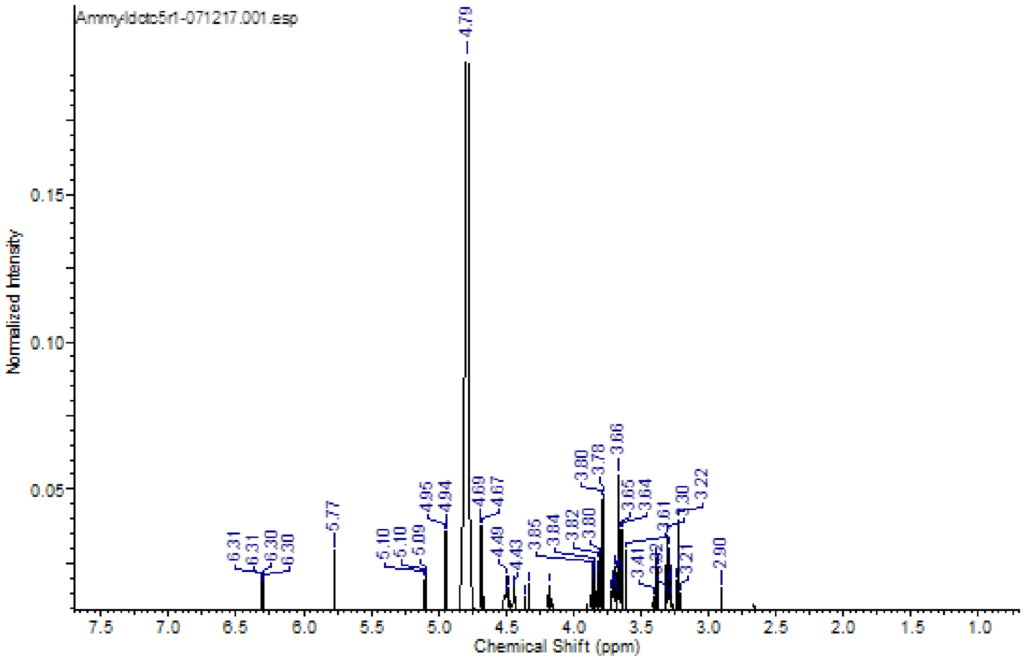

Figure S19. ^{1}H-NMR spectrum of aucubin

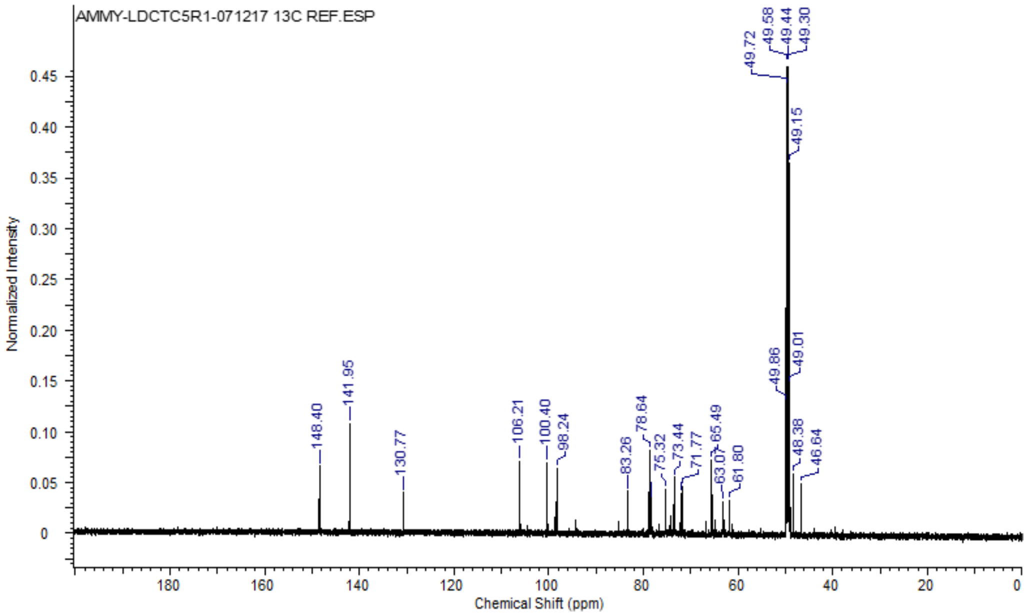

Figure S20. ^{13}C-NMR spectrum of aucubin

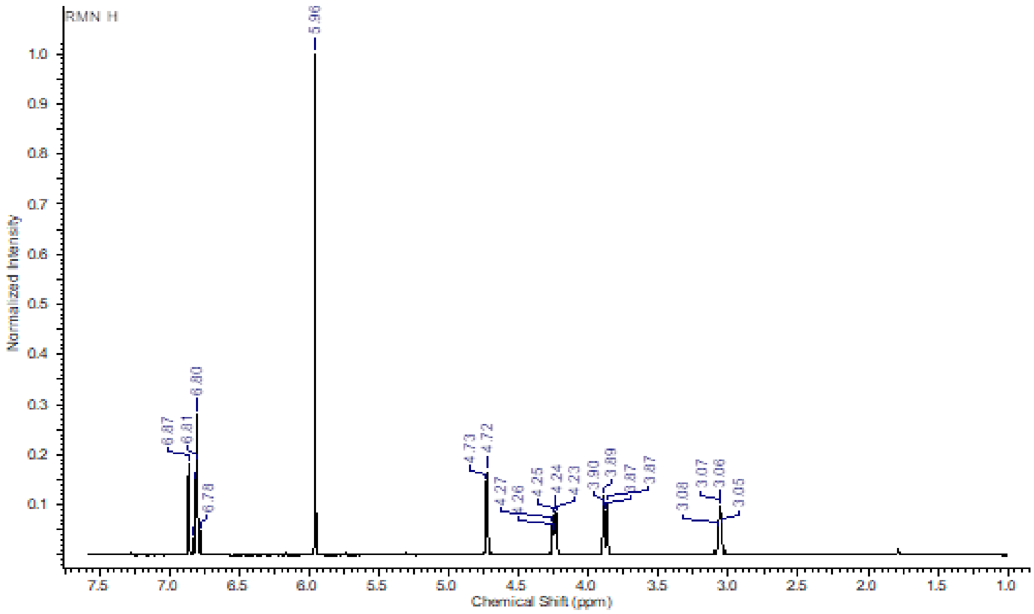

Figure S21. ^{1}H-NMR spectrum of sesamin

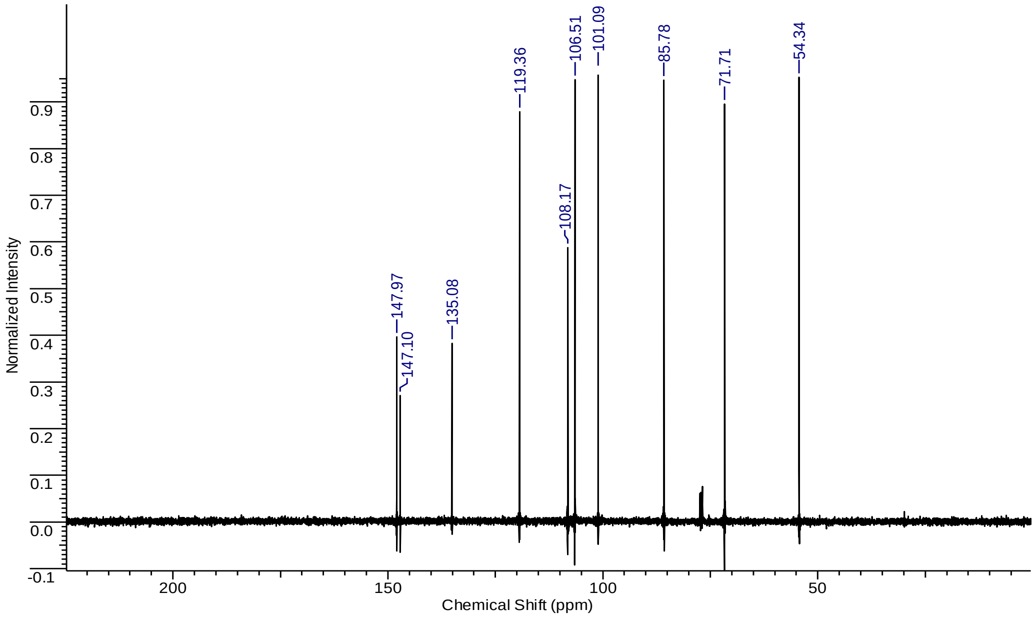

Figure S22. ^{13}C-NMR spectrum of sesamin

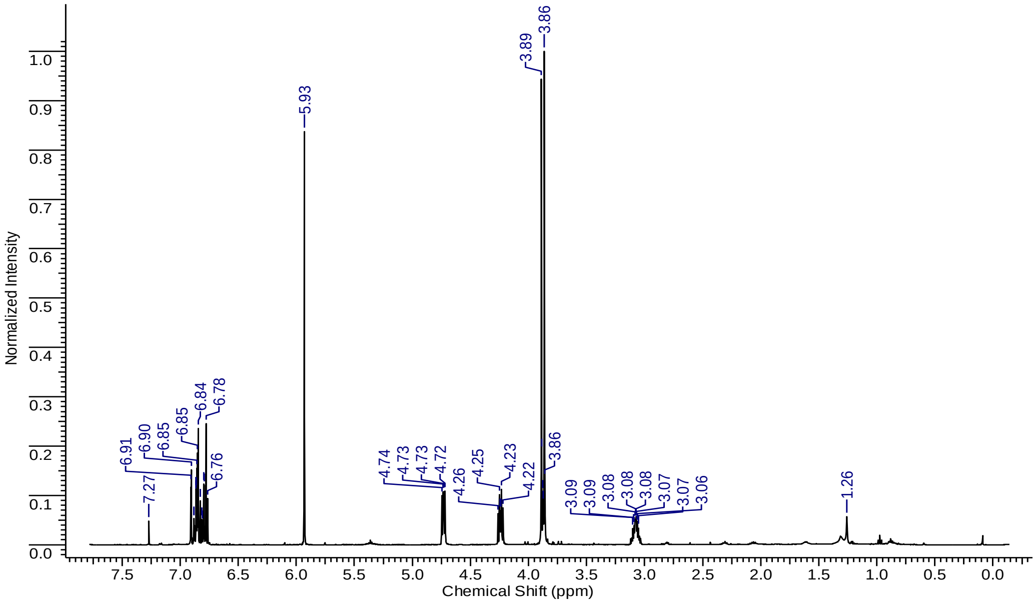

Figure S23. ¹H-NMR spectrum of kobusin

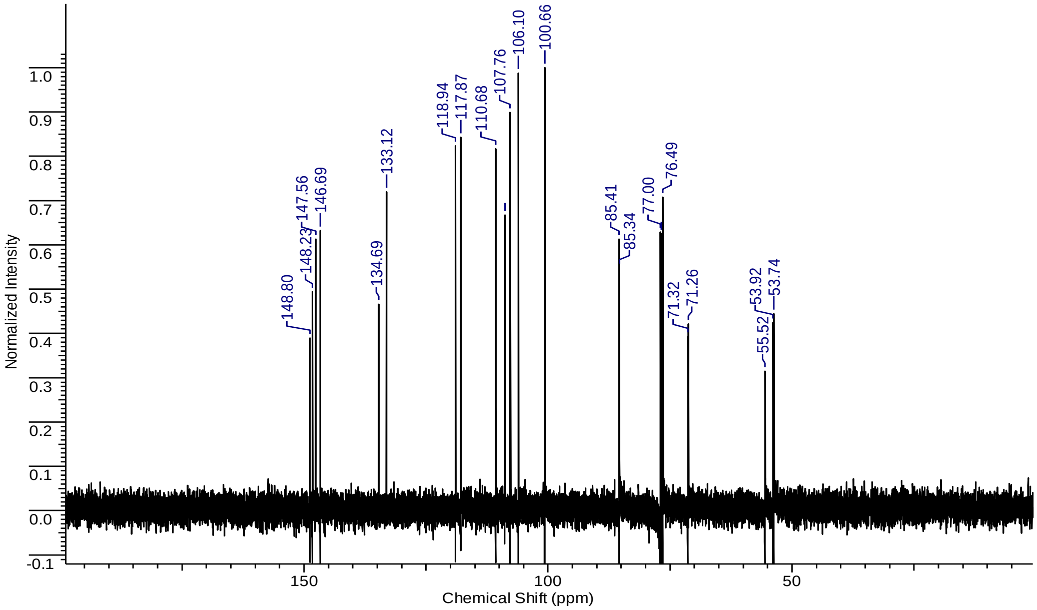

Figure S24. ¹³C-NMR spectrum of kobusin

molecules

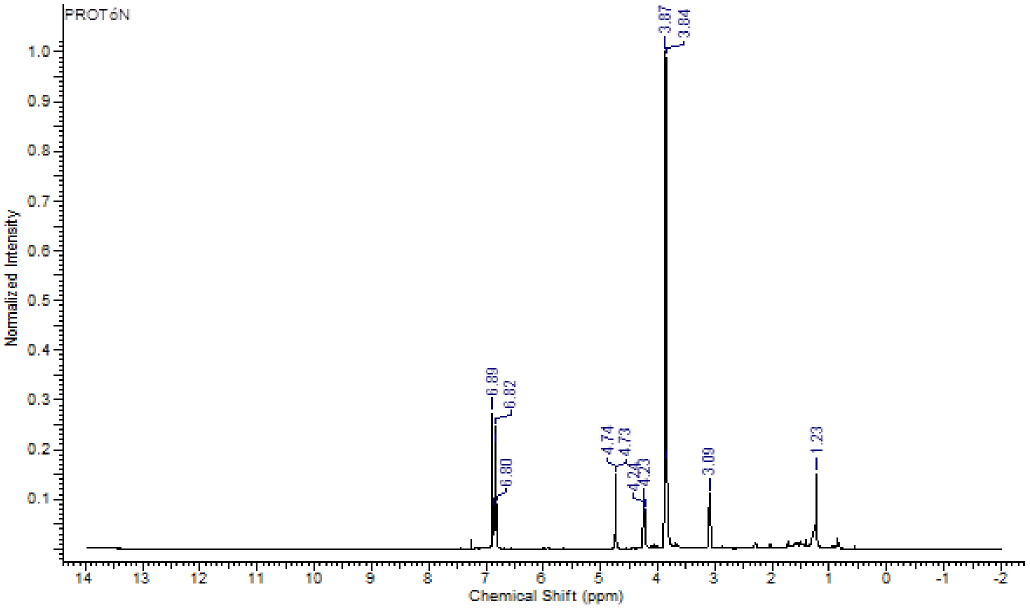

Figure S25. ^{1}H-NMR spectrum of eudesmin

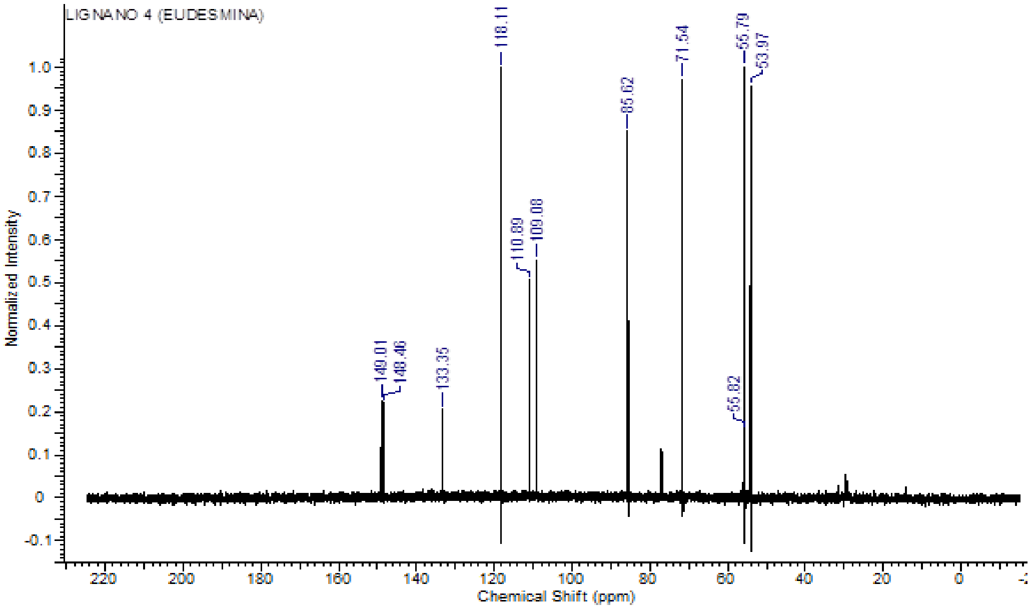

Figure S26. ^{13}C-NMR spectrum of eudesmin

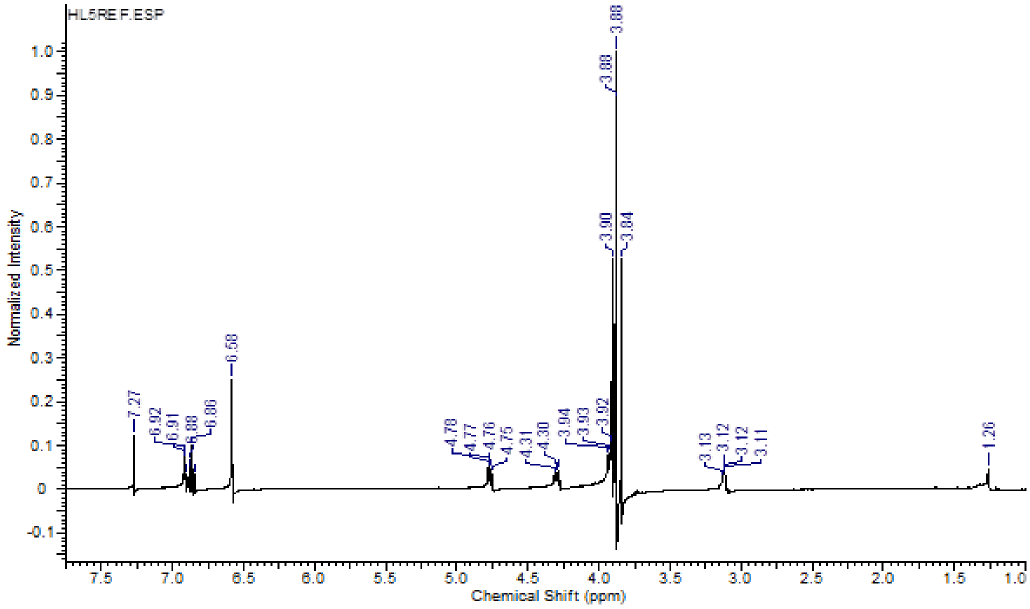

Figure S27. ¹H-NMR spectrum of magnolin

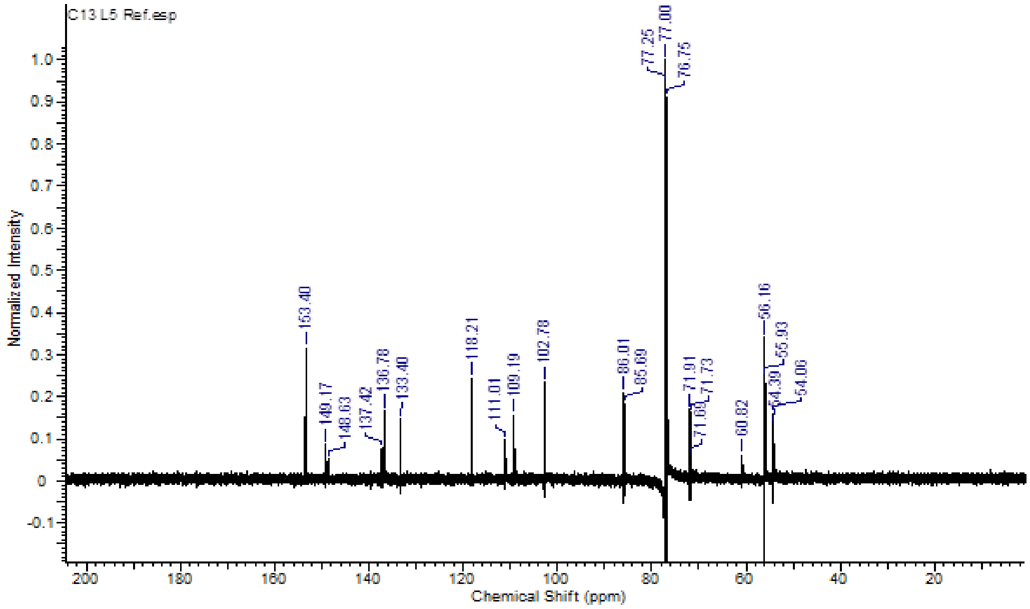

Figure S28. ¹³C-NMR spectrum of magnolin

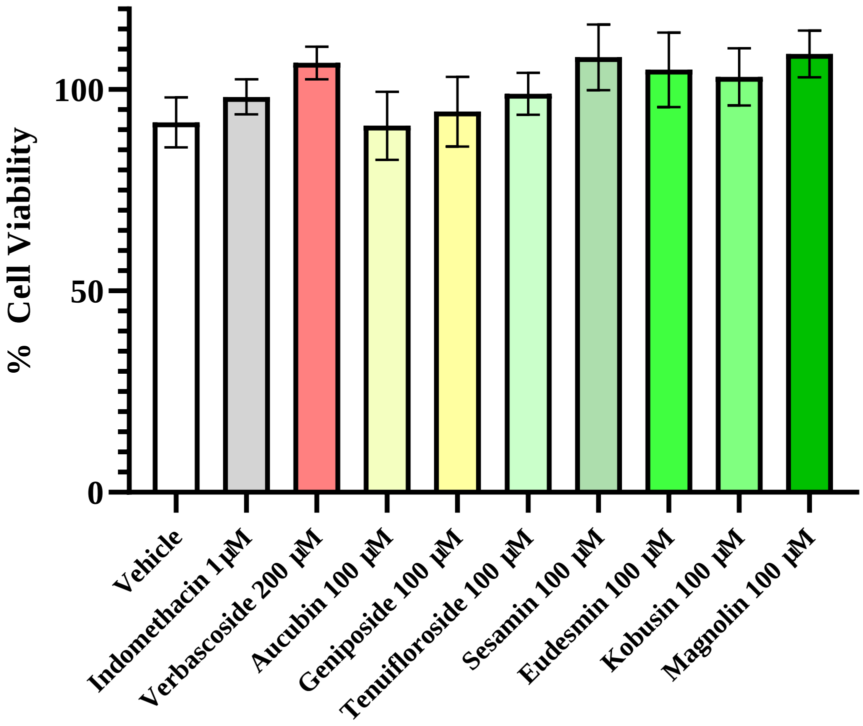

Figure S29. Effect of aucubin, geniposide, verbascoside, tenuifloroside, sesamin, eudesmin, kobusin, and magnolin from *C. tenuiflora* on Raw-blue™ cells viability.

Table S1. Comparative table between calculated inhibition and corroborated inhibition of NF-κB/AP-1 at EC_{50} of isolated compounds from *C. tenuiflora* methanolic extract of aerial parts.

Compound	EC_{50} (µM)	Calculated inhibition at EC_{50} (%)	Corroborated inhibition at EC_{50} (%)
Verbascoside	10.08	27.77	25.40 ± 5.99
Aucubin	0.08	20	28.25 ± 2.73
Geniposide	0.01	28.73	23.18 ±4.12
Tenuifloroside	0.11	26.45	34.78 ± 2.89

Magnolin	3.05	30.48	27.56 ± 7.82
Eudesmin	0.006	28.41	27.30 ± 8.40
Kobusin	2.37	22.72	25.78 ±7.57

Table S2. EC_{50} linearized equations of isolated compounds from *C. tenuiflora* methanolic extract of aerial parts

Compound	Linearized Equation
Verbascoside	y = 0.018X + 0.18152
Aucubin	y = 0.002x + 0.025
Geniposide	y = 0.001x + 0.0174
Eudesmin	y = 0.001005x + 0.0176
Kobusin	y = 0.0521x + 0.022
Magnolin	y = 0.0501x + 0.0164
Tenuifloroside	y = 0.002x + 0.0189
Sesamin	y = 0.0002x + 0.019
